# The extracellular heparan sulfatase SULF2 limits myeloid IFNβ signaling and Th17 responses in inflammatory arthritis

**DOI:** 10.1007/s00018-024-05333-w

**Published:** 2024-08-14

**Authors:** Maarten Swart, Andia N. Redpath, Joy Ogbechi, Ryan Cardenas, Louise Topping, Ewoud B. Compeer, Michael Goddard, Anastasios Chanalaris, Richard Williams, Daniel S. Brewer, Nicola Smart, Claudia Monaco, Linda Troeberg

**Affiliations:** 1 Nuffield Department of Orthopaedics, Rheumatology and Musculoskeletal Sciences, Kennedy Institute of Rheumatology, University of Oxford, Roosevelt Drive, Headington, Oxford, OX3 7FY UK; 2 Department of Physiology, Anatomy & Genetics, University of Oxford, Sherrington Building, Oxford, OX1 3PT UK; 3 Centre for Metabolic Health, Norwich Medical School, University of East Anglia, Bob Champion Research and Education Building, Rosalind Franklin Road, Norwich, NR4 7UQ UK

**Keywords:** Heparan sulfate, Interferon, Inflammation, Macrophage

## Abstract

Heparan sulfate (HS) proteoglycans are important regulators of cellular responses to soluble mediators such as chemokines, cytokines and growth factors. We profiled changes in expression of genes encoding HS core proteins, biosynthesis enzymes and modifiers during macrophage polarisation, and found that the most highly regulated gene was *Sulf2*, an extracellular HS 6-O-sulfatase that was markedly downregulated in response to pro-inflammatory stimuli. We then generated *Sulf2*^+/−^ bone marrow chimeric mice and examined inflammatory responses in antigen-induced arthritis, as a model of rheumatoid arthritis. Resolution of inflammation was impaired in myeloid *Sulf2*^+/−^ chimeras, with elevated joint swelling and increased abundance of pro-arthritic Th17 cells in synovial tissue. Transcriptomic and in vitro analyses indicated that *Sulf2* deficiency increased type I interferon signaling in bone marrow-derived macrophages, leading to elevated expression of the Th17-inducing cytokine IL6. This establishes that dynamic remodeling of HS by *Sulf2* limits type I interferon signaling in macrophages, and so protects against Th17-driven pathology.

**Supplementary Information:**

The online version contains supplementary material available at 10.1007/s00018-024-05333-w.

## Introduction

Inflammation is regulated by a network of cytokines, chemokines and growth factors that control the survival, proliferation, recruitment and signaling responses of innate and adaptive immune cells. Many of these extracellular mediators bind to heparan sulfate (HS) proteoglycans [1], and this interaction controls their localisation, stability, gradient formation and/or signaling [2–4]. For example, binding to extracellular matrix HS is required for the in vivo chemotactic activity of CCL2, CCL4, CCL5 and CXCL4 [5, 6], and regulates the in vivo half-life [7] and bioactivity of IFNγ [8]. While less well-studied, cell surface HS has also been shown to modulate the responses of innate and adaptive immune cells to cytokines, chemokines and growth factors. For example, B cells require cell surface HS to proliferate and differentiate in response to A Proliferation-Inducing Ligand (APRIL) [9, 10] and also to IL21 [11].

HS is a long, structurally diverse polysaccharide that can bind to > 400 protein ligands [1] with a range of affinities, and consequently a broad range of biological effects [12, 13]. Unlike the template-dependent synthesis of nucleic acids and proteins, synthesis of HS is a template-independent process, in which up to 20 enzymes act sequentially in the Golgi apparatus to add alternating uronic acid (either glucuronic acid or iduronic acid) and N-acetylglucosamine residues to the growing HS chain and sulfate it at various positions [13] i.e. glucuronic acid can be 2-O-sulfated (by HS2ST1), while N-acetylglucosamine can be N-sulfated (by NDST1-4), 6-O-sulfated (by HS6ST1-3) or 3-O-sulfated (by HS3ST1-6) during HS biosynthesis [13]. After it has been synthesised and secreted from cells, HS can also be modified in the extracellular environment by heparanase, an enzyme that degrades the HS chains [13], or by the sulfatases SULF1 and SULF2, which specifically remove sulfate groups from the 6-carbon position of N-acetylglucosamine residues [13]. Changes in expression of these biosynthetic and modifying enzymes lead to dynamic changes in HS structure and function during multiple physiological (development [14], wound healing and ageing [15–17]) and pathological (diabetes [18], fibrosis [19, 20], Alzheimer’s disease [21], osteoarthritis [22], cancer [20, 23]) processes.

In myeloid cells, expression of HS sulfotransferases has been shown to change in response to cytokine-induced polarisation [24–26], leading to changes in HS abundance and sulfation [25, 27]. A key study by Gordts et al. [28] showed that myeloid-specific deletion of *Ndst1* sensitised macrophages to type I interferon (IFN) signaling, and exacerbated inflammation in murine models of atherosclerosis and obesity. In vitro mechanistic studies indicated that *Ndst1*-dependent N-sulfation increased IFNβ binding to HS, potentially sequestering the cytokine from its cognate IFN receptors [28]. Subsequent studies showed *Ndst1* can also quench inflammatory responses in dendritic cells (DCs), with deletion of *Ndst1* in DCs increasing antigen presentation and cytotoxic T cell responses in a lung carcinoma model [29].

Here, we systematically profiled changes in expression of the 12 HS core proteins and 25 HS biosynthesis and modifying enzymes in murine bone marrow-derived macrophages (BMDMs) in response to pro- and anti-inflammatory stimuli, and found that the 6-O-sulfatase *Sulf2* was the most highly regulated gene. Using a bone marrow transplantation strategy, we generated chimeric mice with myeloid *Sulf2*-deficiency and examined inflammatory responses in vivo in an antigen-induced arthritis model. This showed that myeloid SULF2 limits inflammation, by suppressing type I interferon signaling and so reducing arthritogenic Th17 responses.

## Materials and methods

### Reagents

IFNγ (BioLegend), IL1, IL2, IL4, IL6, IL13, IL23, macrophage colony-stimulating factor (M-CSF), granulocyte-macrophage colony-stimulating factor (GM-CSF) and transforming growth factor beta (TGFβ) were all purchased from Peprotech, and ultra-pure lipopolysaccharide (LPS) from *Escherichia coli* O111:B4 was purchased from InvivoGen. RT-qPCR primers are detailed in Supplementary Tables S1 and S2, with antibodies used for flow cytometry shown in Supplementary Table S3.

### Primary cell isolation and culture

All experiments were carried out in accordance with the United Kingdom Home Office Animals Scientific Procedures Act 1986 under relevant personal and project licenses.

Single cell suspensions were generated from bone marrow by flushing femurs and tibias with Roswell Park Memorial Institute 1640 medium (RPMI), and adding red blood cell lysis buffer (2 min, room temperature, Sigma). Cells were cultured (7 d) in complete medium [RPMI supplemented with 10% fetal bovine serum (FBS), 1% penicillin/streptomycin, 50 µM 2-mercaptoethanol] supplemented with M-CSF (100 ng/ml, Peprotech) to generate BMDMs), or with GM-CSF (20 ng/ml, Peprotech) to generate bone marrow-derived dendritic cells (BMDCs). BMDMs were replated on day 7, and stimulated for 18 h with IL4 (20 ng/ml, Peprotech) plus IL13 (20 ng/ml, Peprotech), or with LPS (100 ng/ml, InvivoGen) plus IFNγ (100 ng/ml, BioLegend).

Single cell suspensions were generated from joint synovium by digesting opened hindleg knee joints in liberase TL (Roche) and DNase I (Sigma) (30 min, 37 °C), and from lungs by digestion with collagenase IV (Sigma) and DNase I (Sigma) (40 min, 37 °C). Single cell suspensions were generated from spleen and lymph nodes by pressing tissues through a 70 μm cell strainer. All single cell suspensions, except those use for generating chimeras, were treated with red blood cell lysis buffer (2 min, room temperature) before further use.

### Generation of *Sulf2*^+/−^ mice

*Sulf2*^+/−^ mice were generated from the *Sulf2*^tm1a(KOMP)Wtsi^ embryonic stem cell line (RRID: MMRRC_063144-UCD, obtained from the KOMP Repository at University of California, Davis), in which exon 4 of the *Sulf2* gene is flanked by LoxP sites. Embryonic stem cells were injected into blastocysts and resulting chimeras bred to germline transmission of the *Sulf2* tm1a allele, before crossing with Tg(Pgk1-cre)Lni mice [30], available from JAX, to facilitate recombination, and backcrossing to a C57BL/6 background to generate *Sulf2* tm1b allele mice, with a reporter-tagged deletion allele, and missing critical exon 4 of the *Sulf2* gene. The deletion of exon 4 is expected to generate a mis-sense mutation and to cause a premature stop codon in exon 5, which is anticipated to lead to nonsense-mediated decay of the mRNA and loss of protein expression.

### RT-qPCR

RNA was reverse transcribed using a High Capacity Reverse Transcription cDNA kit (Applied Biosystems) after extraction from cultured cells (with RNeasy mini spin-columns, Qiagen), tissues (with TRIzol, Life Technologies), or joints [by homogenisation in a TissueLyser II using a Precellys hard tissue Lysing Kit (Bertin) followed by RNeasy mini spin-columns, Qiagen]. TaqMan Low-Density Array (TLDA) cards (Applied Biosystems) were custom designed using TaqMan primer/probes (Applied Biosystems, Supplementary Table S1), with data acquired on a ViiA Real-Time PCR System (Applied Biosystems). Manual RT-qPCR was carried out using TaqMan primer/probes (Applied Biosystems) with data acquired on a ViiA Real-Time PCR System (Applied Biosystems), or with KiCqStart SYBR Green Primers with data acquired and melt curves examined on a QuantStudio 3 (ThermoFisher Scientific, Supplementary Table S2). Fold change in expression was calculated using the ΔΔCt method.

### Flow cytometry

Cells were stained with Zombie near-infra red (BioLegend) or aqua (ThermoFisher Scientific) fixable live/dead stains (10 min, room temperature), washed in PBS and blocked with anti-mouse CD16/CD32 (10 min, 4 °C, BD Biosciences). For extracellular staining, cells were then incubated with fluorochrome-conjugated antibodies (Supplementary Table S3) in PBS with 1% FBS (4 °C, 30 min), washed twice, and fixed in BD CellFIX (10 min, on ice, BD Biosciences). For intracellular staining, cells were fixed in Foxp3/Transcription factor staining buffer (4 °C, 30 min, Invitrogen), and incubated with fluorochrome-conjugated antibodies in Permeabilisation buffer (4 °C, 30 min, Invitrogen). To quantify intracellular cytokines, cells were stimulated (4 h) with phorbol 12-myristate 13-acetate (PMA, 20 ng/ml) plus ionomycin (1 µg/ml) in the presence of GolgiPlug/GolgiStop (BD Biosciences). Fluorescence was quantified using an LSR-II or Fortessa X20 flow cytometer (BD Biosciences).

### Phagocytosis assays

BMDMs were incubated with 1 μm fluorescent microspheres [Fluoresbrite plain yellow-green particles, multiplicity of infection (MOI) 50, 30 min, Polysciences Inc], fluorescein-conjugated *Escherichia coli* K-12 BioParticles (MOI 5, 15 min, Invitrogen), or fluorescein-conjugated *S. cerevisiae* zymosan A Bioparticles (MOI 5, 20 min, Invitrogen), and phagocytosis quantified by flow cytometry. Efferocytosis was quantified by incubating BMDMs with Jurkat T cells (MOI 2, 45 min) which had been labelled with calcein-acetoxymethyl (2 h, 37 °C, ThermoFisher Scientific) and exposed to UV light (305 nm, 2.5 h) to induce apoptosis. As a negative control for all phagocytosis assays, particles were also incubated with BMDMs on ice.

### Generation of bone marrow chimeras and antigen-induced arthritis (AIA)

Recipient 8-week old wild-type (WT) C57BL/6 mice were irradiated (two doses of 5.5 Gy at a 4 h interval in a Gulmay X-ray irradiator), and injected 2 h later in the tail vein with bone marrow cells (5 million/mouse) from 8-week old WT or *Sulf2*^+/−^ donors. Seven weeks later, mice were subcutaneously injected with methylated bovine serum albumin (mBSA, 100 µg, Sigma) emulsified 1:1 with Complete Freund’s adjuvant (BD Biosciences). Arthritis was induced 3 weeks later by intra-articular tibiofemoral injection of mBSA (100 µg in PBS, right knee) or PBS control (left knees). Knee swelling was measured daily for 7 days after intra-articular injection using a calliper, and pain assessed by measuring weight bearing on mBSA-injected compared to PBS-injected knees using a dynamic weight-bearing 2.0 chamber (Bioseb). Mice were sacrificed after 7 days, and immune infiltration and total histological score, composed of the sub-synovium, synovium and bone marrow density scores, calculated from blinded scoring [31]. Immunohistochemical analysis of pSTAT1 (Y701) (D4A7, Cell Signaling, followed by goat anti-rabbit Alexa 488, ThermoFisher Scientific) and CD206 (C068C2, BioLegend, followed by goat anti-rat Alexa 568, ThermoFisher Scientific) abundance was done following antigen retrieval in Tris-EDTA. Fluorescent signal (mean fluorescence per cell cluster x area) was quantified for at least 700 cell clusters per genotype using a BioTek Cytation 7 imaging reader and Gen5 v 3.14 software. To analyse T cell subsets, single cell suspensions from knee joints and lymph nodes were stimulated for 4 h with PMA (20 ng/ml) and ionomycin (1 mg/ml) in the presence of protein transport inhibitors, before CD3^+^, CD4^+^ and CD8^+^ subsets were analysed by flow cytometry.

### Bulk RNASeq

RNA was isolated (RNeasy Micro Kits, Qiagen) from single cell suspensions prepared from knee joints of WT and *Sulf2*-deficient bone marrow chimera mice sacrificed 7 days after induction of arthritis. Oxford Genomics Centre prepared bulk RNA libraries using an NEBNext Single Cell/Low Input RNA Library Prep Kit for Illumina (New England Biolabs) and sequenced the samples at a depth of 25 million reads per sample on a NovaSeq6000 (Illumina). PolyA transcripts were analysed with the Nextflow RNA-Seq pipeline [32], using STAR for alignment with the GRCm38 reference murine genome. Deconvolution of cell types was performed with MuSIC [33], utilising single cell data from Park et al. [34]. DeSeq2 was used for differential gene expression analysis, with correction for multiple comparisons carried out using the Benjamini and Hochberg method. Functional enrichment analysis was performed using gProfiler2 (v0.2.0) utilising the Kyoto Encyclopedia of Genes and Genomes (KEGG) pathway database [35] for terms. The gSCS (Set Counts and Sizes) correction method was used to determine significantly enriched pathways with significance *p* < 0.05. Differentially regulated genes were manually compared with the Interferome database (interferome.org, [36]), and Homer was used to analyse promoter regions (8 bp motifs, -400 to -100 base pairs upstream of transcriptional start sites) of differentially expressed genes [37].

### Analysis of type I interferon signaling

For analysis of STAT1 phosphorylation, BMDMs from WT and *Sulf2*^*+/−*^ mice were stimulated with murine IFNβ (50 ng/ml, 30 min, Peprotech) and phospho-STAT1 (Y701) (D4A7, Cell Signaling) and STAT1 (#9172, Cell Signaling) quantified by immunoblotting analysis on an Odyssey CLx fluorescent imaging system (LI-COR Biosciences) using Image Studio software (ver 4.0, LI-COR Biosciences).

For analysis of interferon-regulated gene expression, BMDMs from WT and *Sulf2*^*+/−*^ mice were stimulated with murine IFNβ (50 ng/ml, 4 h, Peprotech) and expression of *Ccl5*, *Ccl7*, *Tlr3* and *Il6* quantified by RT-qPCR relative to *Gapdh*.

### Primeflow analysis of *Sulf2* mRNA expression in blood, bone marrow, spleen, and lung

The PrimeFlow RNA assay (Invitrogen) was used according to the manufacturer’s protocol to assess *Sulf2* mRNA expression in various cell types. Isolated cells were surface-stained, permeabilised, fixed, and hybridised with a target probe set (2 h, 40 °C). After hybridisation of pre-amplifying primers (1.5 h, 40 °C) and amplifying primers (1.5 h, 40 °C), samples were incubated with labelled probes (1 h, 40 °C), stained for immune cell markers (CD45, CD3, CD19, Ly6C, Ly6G in blood and bone marrow; CD45, CD3, CD19, F4/80, CD11b, Ly6C, Ly6G, CD11c, CD64, MHC-II in spleen and lung) and fluorescence quantified using a Fortessa X20 flow cytometer (BD Biosciences).

### Toll-like receptor (TLR) signaling

Macrophages were stimulated with the TLR agonists IFNγ, LPS, IFNγ + LPS (all at 100 ng/ml), fibroblast-stimulating lipopeptide-1 (FSL-1, 100 ng/ml), 10 ng/ml polyinosinic: polycytidylic acid [poly (I: C)], or 100 ng/ml N-palmitoyl-S-[2,3-bis(palmitoyloxy)-(2RS)-propyl]-[R]-cysteinyl-[S]-seryl-[S]-lysyl-[S]-lysyl-[S]-lysyl-[S]-lysine (Pam3CSK4) for 6 h and IL6 secretion measured by ELISA (R&D Systems).

### Inflammasome activation

Macrophages were stimulated with 100 ng/ml LPS (100 ng/ml, 6 h) with nigericin (5 µM) added for the last 2 h of culture. Cell death was assessed using the CytoTox 96 Cytotoxicity assay (Promega) and IL1β secretion measured by ELISA (Invitrogen).

### Antigen uptake and presentation

Antigen uptake was measured by flow cytometry analysis of BMDMs incubated with Alexa647-labelled ovalbumin (OVA, 0.1 mg/ml, 1 h, 37 °C, Molecular Probes). To assess antigen presentation, CD4^+^ T-cells were enriched from the spleen of OT-II mice using the MojoSort mouse CD4^+^ T-cell isolation kit (BioLegend) and labelled with CellTrace Violet (5 µM, 20 min, 37 °C, Invitrogen). Labelled CD4^+^ T-cells were co-cultured (4 d) with WT or *Sulf2*^+/−^ antigen-presenting cells (BMDMs or BMDCs) activated with LPS (100 ng/ml LPS, 4 h, InvivoGen). As a source of antigen, OVA (0.1 mg/ml, InvivoGen) was added to antigen-presenting cells (1 h, 37 °C) before LPS-activation, or OVA peptide 323–339 (10 µg/ml, Peptides International) was added during co-culture of antigen-presenting cells with CD4^+^ T-cells. CD4^+^ T-cell proliferation and expression of CD25 were analysed by flow cytometry, with division index, proliferation index and percent divided cells determined using the FloJo proliferation tool.

### Cytotoxicity assay

A CytoTox 96 Cytotoxicity assay (Promega) was performed according to manufacturer’s instructions. This assay detects release of lactate dehydrogenase from damaged cells.

### Statistical analysis

Generation of heatmaps and principal component analysis (PCA) analysis was performed in R (version 3.5.2) and R studio (version 1.1.453). For PCA analysis, the prcomp function was used, without any maximum number of ranks and without clustering. The samples were then plotted on the obtained and rotated principal components, together with ellipsoids indicating 95% confidence around the centroids of the data groups. All other data were analysed using GraphPad Prism (version 8.4.1). Data were analysed for normality using the D’Agostino-Pearson test, and for statistical significance using relevant tests as detailed in the figure legends. Data are presented as mean ± SEM or median ± IQR where appropriate.

## Results

### Macrophage expression of HS core proteins and modifying enzymes was regulated by polarisation in vitro

**Fig. 1:**
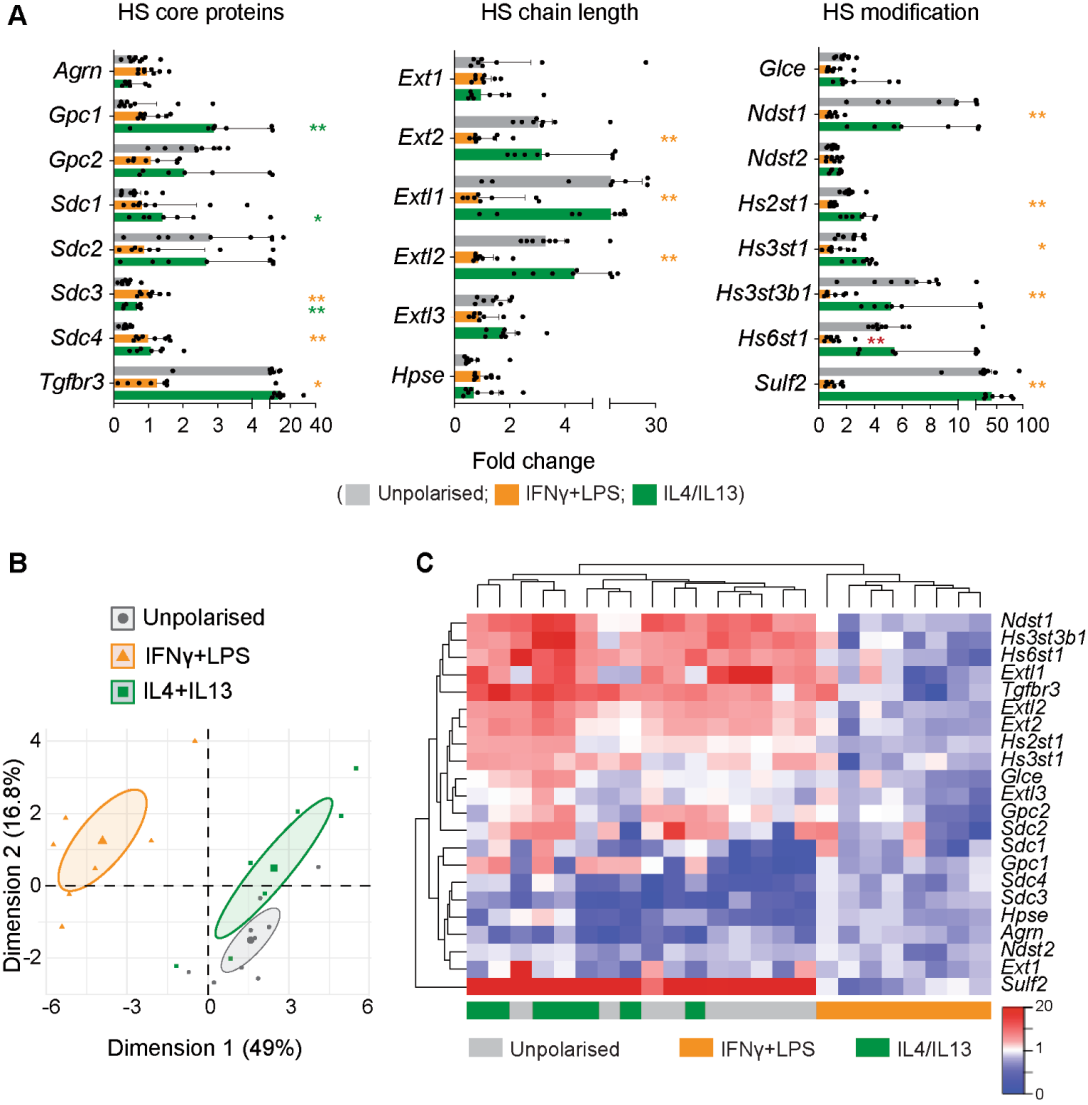
Expression of HS core proteins and modifying enzymes was downregulated by IFNγ + LPS. (**A-C**). Bone marrow-derived macrophages were polarised in vitro with IFNγ + LPS or IL4 + IL13 (18 h, *n* = 7–9 mice per group). mRNA expression of the 37 HS core proteins and modifying enzymes was quantified by RT-qPCR using custom-designed TaqMan low-density array cards, and expressed relative to the geometric mean of the housekeeping genes *18s*, *Gapdh*, and *Rplp0*, and to the average expression in IFNγ + LPS-polarised cells. (**A**). Fold change in expression of the 22 detectably-expressed HS core proteins and modifying enzymes (median ± IQR, analysed by Kruskall-Wallis test followed by multiplicity correction using the two-stage step-up method of Benjamini, Krieger and Yekutieli. *, *P* < 0.05; **, *P* < 0.01, shown in orange for IFNγ + LPS and in green for and IL4 + IL13). (**B**). PCA analysis of differentially expressed genes, with ellipses indicating the 95% confidence interval. (**C**). Heatmap showing changes in expression of the 22 detectably-expressed HS genes. For PCA analysis, the prcomp function was used, without any maximum number of ranks and without clustering. The samples were then plotted on the obtained and rotated principal components, together with ellipsoids indicating 95% confidence around the centroids of the data groups

We profiled the expression of genes encoding the 37 HS core proteins and modifying enzymes in BMDMs, and found that they expressed detectable levels of 22 of these genes (8 of the possible 12 core proteins and 14 of the possible 25 modifying enzymes). Polarisation of the cells in vitro with IFNγ + LPS or IL4 + IL13 significantly altered expression of 60% of the expressed genes (Fig. 1A), with 3 distinct groups on a PCA analysis (Fig. 1B).

Polarisation with IFNγ + LPS generated a distinct gene expression profile (Fig. 1B-C), with significantly altered expression of three core proteins (increased *Sdc3* and *Sdc4*, and reduced *Tgfbr3* compared to unpolarised BMDMs) and lower expression of many of the HS modifying enzymes (Fig. 1A). At least one gene from each of the groups of HS modifying enzymes was significantly downregulated by IFNγ + LPS, with significant changes in expression of genes that control HS chain elongation (*Ext2*, *Extl1*, *Extl2*), N-sulfation (*Ndst1*), 2-O-sulfation (*Hs2st1*), 3-O-sulfation (*Hs3st1*, *Hs3st3b1*) and 6-O-sulfation (*Hs6st1*, *Sulf2*) (Fig. 1A).

Polarisation with IL4 + IL13 had limited effects, with altered expression of only three core proteins (increased *Gpc1*, *Sdc1*, *Sdc4* compared to unpolarised BMDMs, Fig. 1A-C).

The most strongly regulated gene was *Sulf2*, a sulfatase that removes 6-O sulfate groups from N-acetylglucosamine residues of HS. Expression of this gene was markedly down-regulated in response to IFNγ + LPS treatment (25-fold compared to unpolarised BMDMs, Fig. 1A).

### *Sulf2*-deficiency altered macrophage polarisation in vitro

**Fig. 2:**
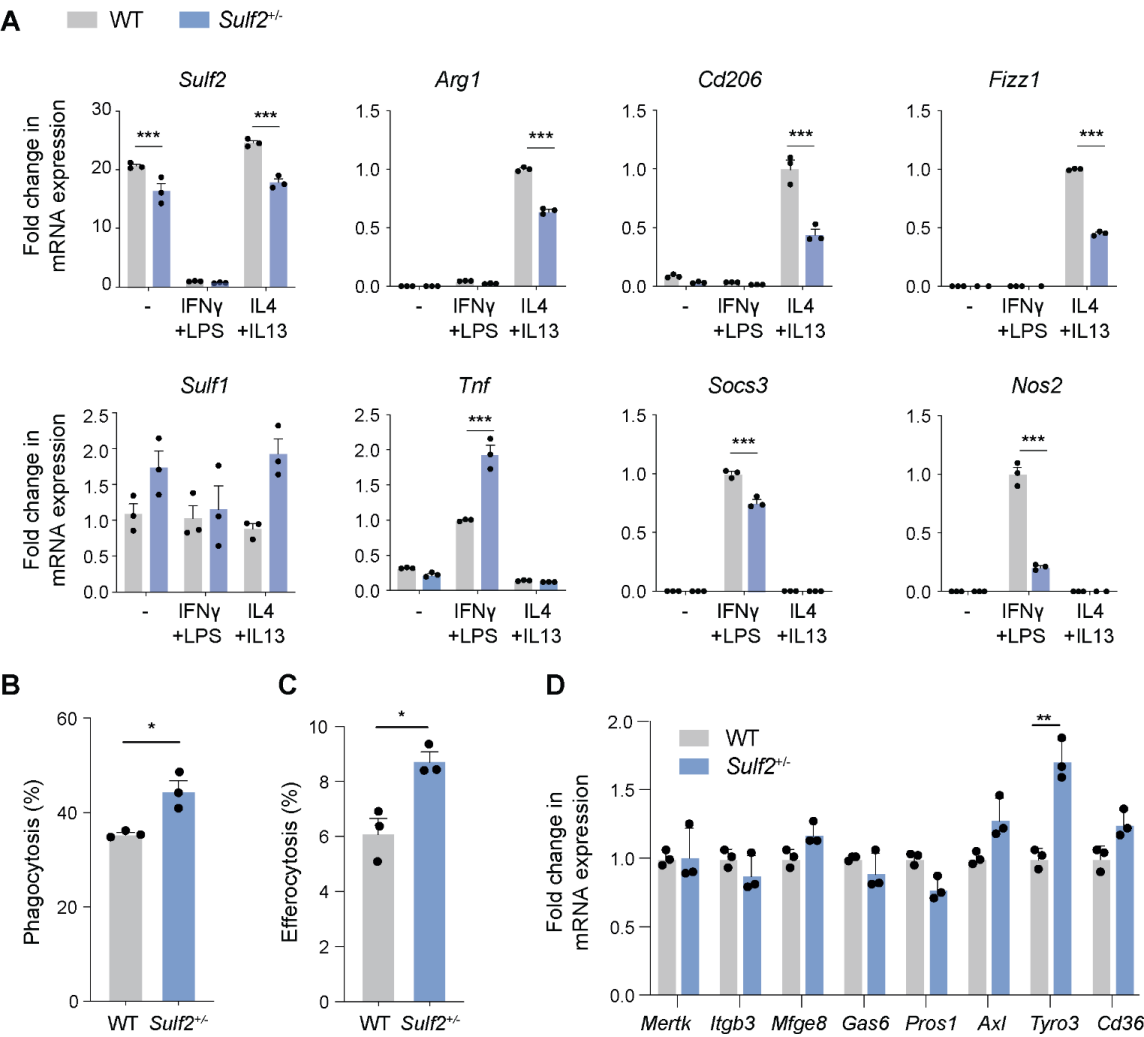
Macrophage polarisation and phagocytosis was modulated by *Sulf2*. BMDMs from WT and *Sulf2*^*+/-*^ mice were stimulated with IFNγ + LPS or IL4 + IL13 (18 h). (**A**). Fold change in gene expression was quantified relative to *Rplp0* (*n* = 3, mean ± SEM, analysed by two-way ANOVA with Bonferroni’s correction for multiple comparisons. ***, *P* < 0.001). (**B-C**). Cells were incubated with fluorescent polystyrene beads at 50 MOI (B) or apoptotic cells at 1 MOI, and the percentage of percentage of fluorescence positive cells determined by flow cytometry (*n* = 3, mean ± SEM, analysed by two-way Student’s *t*-tests. *, *P* < 0.05). (**D**). Fold change in gene expression was quantified relative to *Rplp0* and to WT cells (n = mean ± SEM, analysed by multiple unpaired *t*-tests, followed by multiplicity correction using the two-stage step-up method of Benjamini, Krieger and Yekutieli, **, q < 0.01)

We observed perinatal lethality in *Sulf2*^−/−^ mice, as has been reported by others [38, 39], so we utilised *Sulf2*^+/−^ BMDMs to investigate the impact of partial *Sulf2* deficiency in macrophages. Given that unpolarised BMDMs expressed high levels of *Sulf2*, we investigated the effect of *Sulf2*-deficiency on polarisation of these cells (Fig. 2A). IL4 + IL13-treated *Sulf2*^*+/−*^ BMDMs expressed significantly lower levels of *Arg1*, *Cd206* and *Fizz1*, suggesting that *Sulf2*-deficiency may reduce polarisation to an anti-inflammatory phenotype. IFNγ + LPS-treated *Sulf2*^*+/−*^ BMDMs expressed significantly higher levels of the prototypic pro-inflammatory marker *Tnf* (i.e. TNFα) and reduced levels of *Socs3*, a suppressor of cytokine signaling (Fig. 2A), indicating a potentially more inflammatory phenotype, although these cells also expressed lower levels of *Nos2*.

Potential mechanisms leading to these changes in polarisation were investigated. There was no evidence for a change in IL6 release in response to IFNγ, LPS, poly(I: C), FSL-1 or Pam3CSK4, and a small effect size increase in IL6 release in response to IFNγ + LPS (Fig. S1A), indicating TLR signaling was not markedly impacted by *Sulf2*-deficiency. Similarly, there was no significant difference in pyroptotic cell death and IL1β release in response to LPS + nigerycin was unaffected (Fig. S1B), indicating unaltered inflammasome activation in *Sulf2*-deficient BMDMs.

### *Sulf2*-deficiency increased phagocytosis in vitro

To further assess the phenotype of *Sulf2*-deficient BMDMs, we profiled their phagocytic and antigen-presenting abilities.

Mean phagocytosis of polystyrene beads was increased by 25% (Fig. 2B, *P* < 0.05) and phagocytosis of apoptotic cells was increased by 43% (Fig. 2C, *P* < 0.05) in *Sulf2*-deficient cells. This was accompanied by significantly increased expression of the efferocytosis receptor *Tyro3*, but not *Mertk*, *Axl* or *Cd36* (Fig. 2D). Expression of integrin αvβ3 (*Itgb3*) and the efferocytosis bridging molecules *Gas6* and *Pros1* was similarly unaffected.

There was no evidence that in vitro uptake of OVA antigen and cell surface expression of costimulatory molecules (MHC-I, CD80, CD86) was altered in *Sulf2*-deficient BMDMs and BMDCs, but expression of MHC II was reduced with a small effect size in *Sulf2*^+/−^ BMDCs (Fig. S2). Similarly, there was no evidence that *Sulf2*-deficiency had an effect on in vitro presentation of OVA to CD4^+^ T cells by BMDCs, with unaltered CD4^+^ T cell proliferation and expression of CD25 (Fig. S3).

### Myeloid *Sulf2* deficiency increased joint damage and inflammation in vivo

*Sulf2* is highly expressed by myeloid cells (Fig. S4), but it is also expressed by multiple other cell types [40]. To study the function of myeloid *Sulf2*, we thus generated chimeric mice by irradiating WT animals and reconstituting their myeloid population with BMDMs isolated from either WT or *Sulf2*^+/−^ donors. The effect of *Sulf2*-deficiency on myeloid function was then assessed using the antigen-induced arthritis (AIA) model, in which immunisation and boosting of mice with mBSA leads to myeloid and lymphoid cell-dependent joint inflammation and damage in mice [31].

**Fig. 3:**
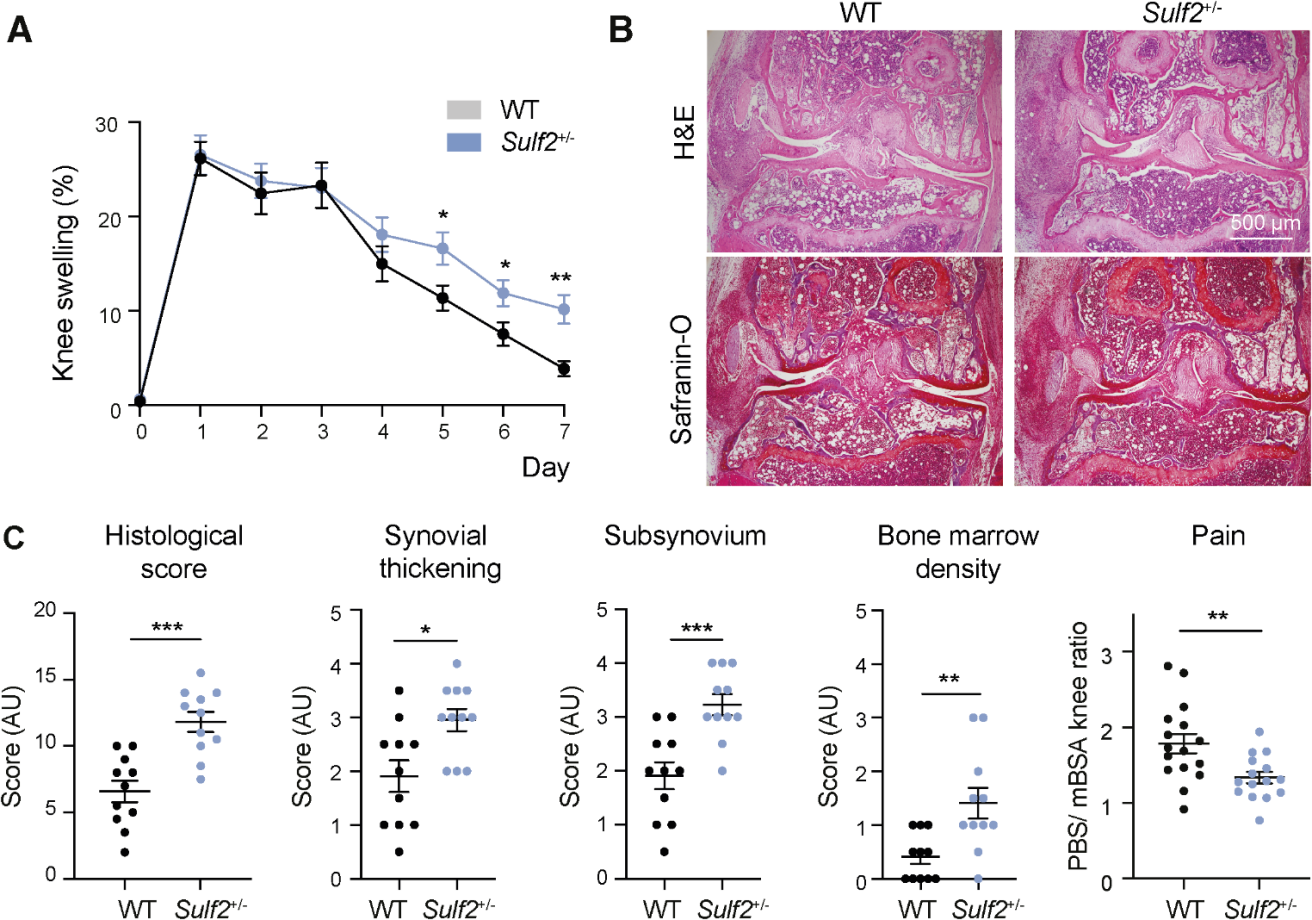
Swelling and AIA histology scores were increased at day 7 in *Sulf2*-deficient bone marrow chimera mice. WT and *Sulf2*^*+/-*^ bone marrow chimeric mice (*n* = 11–15) were immunised with mBSA in CFA (100 µg) and arthritis was induced 3 weeks later by intra-articular tibiofemoral injection of mBSA (100 µg, right knee). Mice were sacrificed 7 days later and disease severity in the knees was visualised histologically following safranin-O and H&E staining. (**A**). Knee swelling (*n* = 15) was measured daily for 7 days after intra-articular injection using a calliper (mean ± SEM, analysed by a multiple unpaired *t*-tests, followed by multiplicity correction using the two-stage step-up method of Benjamini, Krieger and Yekutieli, **, q < 0.01. *, q < 0.05). (**B**). Representative histology at day 7. (**C**). Histology of the joint (4x magnification, *n* = 11) was examined to calculate the total histological score, synovium and subsynovium thickness, and bone marrow density. Pain was assessed by measuring weight bearing using a Bioseb weight-bearing chamber, with data presented as the ratio of time spent on the PBS-injected limb relative to the mBSA-injected limb (mean ± SEM, analysed by a two-tailed Mann-Whitney u test. *, *P* < 0.05; **, *P* < 0.01; ***, *P* < 0.001)

WT and *Sulf2*^+/−^ chimeric mice developed similar acute inflammation in the first 4 days after intra-articular injection with mBSA, with no significant difference in initial knee swelling (Fig. 3A) or histological joint damage (Fig. S5). However, the resolution of inflammation was significantly impaired in *Sulf2*^+/−^ chimeric mice, with knee swelling significantly elevated at days 5–7. This was accompanied by increased joint damage in the *Sulf2*^+/−^ chimeric mice, with a significantly increased total histological score, synovial and subsynovial thickening and bone marrow density at day 7 (Fig. 3B-C). Paradoxically, *Sulf2*^+/−^ chimeric animals exhibited less pain at day 7 (Fig. 3C).

### Myeloid *Sulf2* deficiency increased Th17/Treg ratio in knees

The increased joint swelling in *Sulf2*^+/−^ chimeric mice was not explained by increased immune cell infiltration, as flow cytometry showed there was no significant difference in the abundance or frequency of myeloid or adaptive immune cell types in the knees of WT and *Sulf2*^+/−^ chimeric mice 2 or 7 days after initiation of AIA (Fig. S6).

**Fig. 4:**
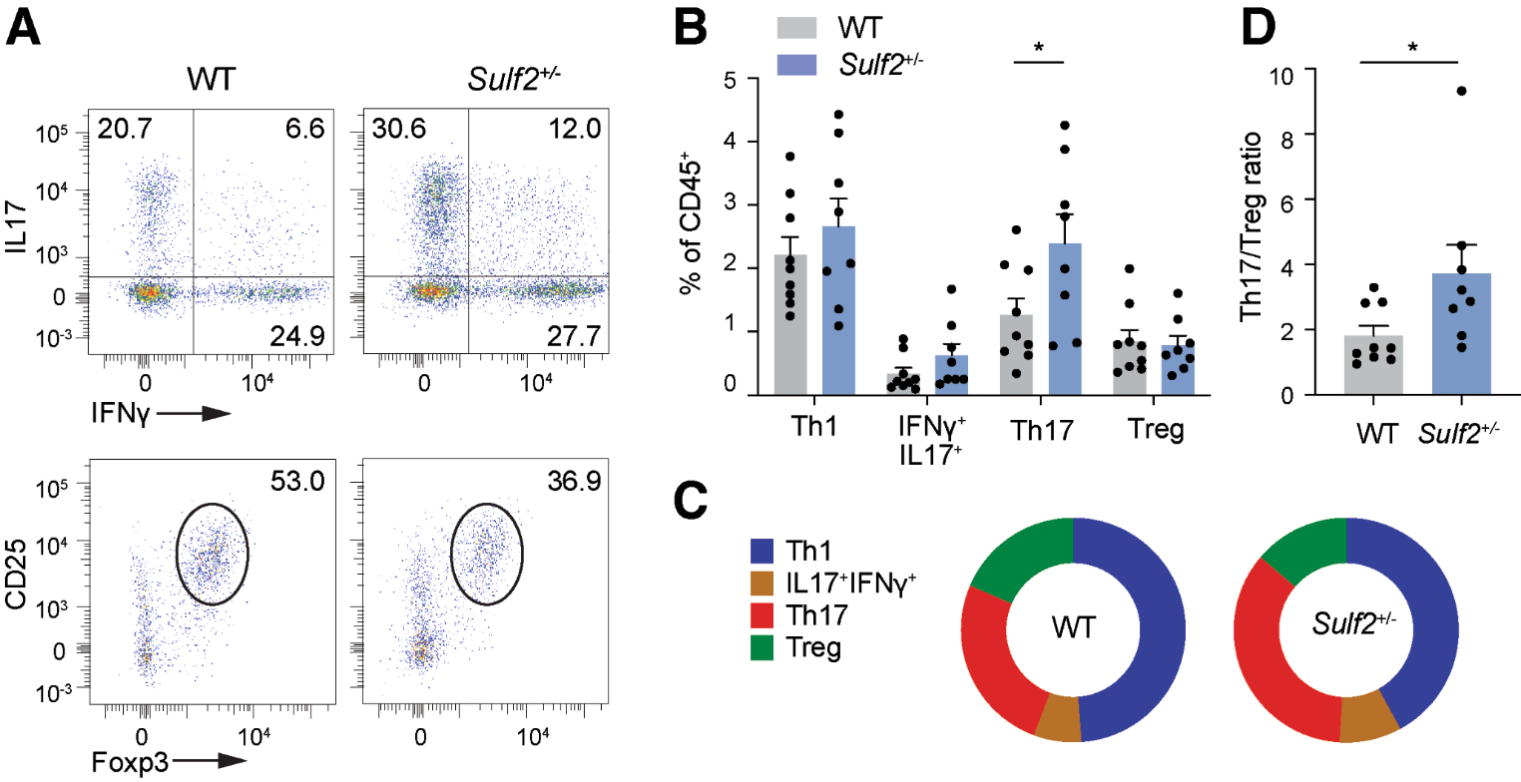
The Th17/Treg ratio was increased in the knee joints of *Sulf2*-deficient mice at day 7 of AIA. Single cell suspensions were isolated from knees of WT and *Sulf2*^*+/−*^ bone marrow chimeric mice (*n* = 9) 7 days after initiation of arthritis, and stimulated for 4 h with PMA (20 ng/ml) and ionomycin (1 µg/ml) in the presence of protein transport inhibitors. CD3^+^, CD4^+^ and CD8^+^ T-cell subsets were analysed by flow cytometry. (**A**). Representative dot plots for Th17 (IFNγ^+^IL17^+^) and Treg (CD25^+^Foxp3^+^) subsets in AIA knees. (**B**). The abundance of T cell subsets (as a percentage of CD45^+^ cells) was calculated (mean ± SEM, analysed by ANOVA with Sidak’s correction for multiple comparisons. *, *P* < 0.05). (**C**). Graphical representation of data in (**B**). (**D**). The Th17/Treg ratio was calculated from data in (**B**) (mean ± SEM, analysed by a two-tailed Student’s *t*-test. *, *P* < 0.05)

While the overall number of CD4^+^ T cells was not altered (Fig. S6), closer examination of this compartment revealed a significant increase in Th17 abundance in the knees of *Sulf2*^+/−^ chimeric mice on day 7 of AIA (Fig. 4A-B), leading to a 2-fold increase in the Th17/Treg ratio (Fig. 4C-D). This shift in the Th17/Treg ratio was not observed in the inguinal lymph nodes (Fig. S7), indicating it was a local rather than a systemic effect.

### Type I interferon signaling was elevated in *Sulf2*-deficient macrophages

**Fig. 5:**
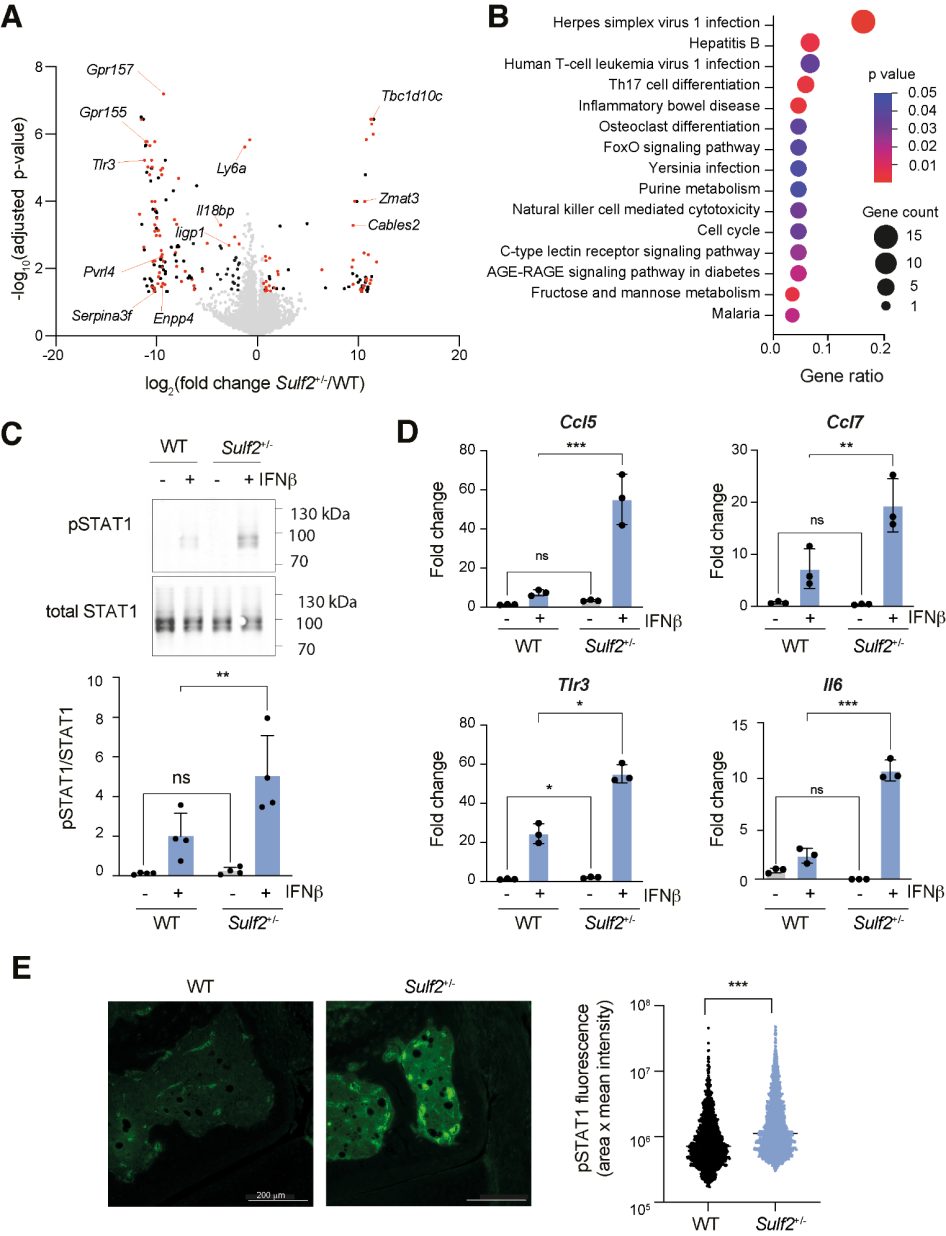
Type I interferon signaling was increased in in *Sulf2*-deficient macrophages. (**A-B**). Single cell suspensions were isolated from knees of WT and *Sulf2*^*+/−*^ bone marrow chimeric mice (*n* = 3) 7 days after initiation of arthritis and bulk RNA sequencing performed. (**A**). The volcano plot shows differentially expressed genes (fold change in *Sulf2*^+/−^ compared to WT) identified by DeSeq2, with significantly altered type I interferon-responsive genes [36] labelled in red, other significantly altered genes in black, and non-significantly altered genes in grey. (**B**). The top 15 KEGG identifiers enriched in differentially expressed genes are shown, indicating their P-value and gene ratio (fraction of the differentially-expressed genes in each KEGG identifier). (**C**). BMDMs from WT and *Sulf2*^*+/-*^ mice were stimulated with IFNβ (50 ng/ml, 30 min) and phosphorylation of STAT1 as a fraction of total STAT1 quantified by immunoblotting (*n* = 4 biological replicates per group, mean ± SD, analysed by a 2-way ANOVA with Sidak’s correction for multiple comparisons. **, *P* < 0.01). (**D**). WT and *Sulf2*^*+/−*^ BMDMs (*n* = 3 biological replicates per group) were stimulated with IFNβ (50 ng/ml, 4 h) and expression of *Ccl5*, *Ccl7*, *Tlr3* and *Il6* quantified by RT-qPCR relative to *Gapdh* (*n* = 3 biological replicates per group, mean ± SD, analysed by a 2-way ANOVA with Sidak’s correction for multiple comparisons. *, *P* < 0.05; **, *P* < 0.01; ***, *P* < 0.001). (**E**). pSTAT1 staining was also increased in knee joints of *Sulf2*^*+/−*^ bone marrow chimeric mice on day 7 of AIA (*n* = 3 per genotype, analysed by two-tailed Mann-Whitney u test. ***, *P* < 0.001; ns, not significant)

To gain insight into the mechanism underlying increased Th17 generation in *Sulf2*^+/−^ chimeric mice, we conducted bulk RNA sequencing on cells isolated from knees of WT and *Sulf2*^+/−^ chimeric mice 7 days after initiation of AIA (Fig. 5A). 199 genes were found to be significantly differentially expressed between WT and *Sulf2*^+/−^ chimeric mice (76 up-regulated and 123 down-regulated; Padj < 0.05 by DESeq2, Table S4). Deconvolution of the data with MuSIC [33] estimated that the vast majority of sequences (> 85%) originated from macrophages (Fig. S8). KEGG pathway enrichment analysis of the significantly differentially expressed genes was indicative of altered type I interferon signaling, with significantly enriched KEGG identifiers including “Herpes simplex virus infection 1” (*P* = 1.4 × 10^− 4^, gene ratio = 0.16), “Hepatitis B” (*P* = 3.2 × 10^− 3^, gene ratio = 0.07) and “Human T-cell leukemia virus 1 infection” (*P* = 3.6 × 10^− 3^, gene ratio = 0.07) (Fig. 5B). Subsequent comparison with the Interferome database [36] indicated that 111 of the 199 differentially expressed genes (56%) are known to be type I interferon-responsive genes (marked in red on Fig. 5A). Analysis of the promoter region of differentially regulated genes indicated that 42% contain recognition sites for the type I interferon-related transcription factor ELF4 [41] (*P* = 1 × 10^− 2^) and 11% contain recognition sites for IRF3 [42] (*P* = 1 × 10^− 2^), both transcription factors known to bind to interferon-stimulated response elements.

To validate these findings, WT and *Sulf2*^+/−^ BMDMs were stimulated in vitro with IFNβ and phosphorylation of STAT1 quantified. This showed a significant increase in pSTAT1/STAT1 in *Sulf2*^+/−^ BMDMs (Fig. 5C). Similarly, *Sulf2*^+/−^ BMDMs expressed significantly higher levels of the interferon-stimulated genes *Ccl5*, *Ccl7*, *Tlr3* and *Il6* in response to IFNβ stimulation (Fig. 5D). pSTAT1 was also significantly increased in knee sections from *Sulf2*^+/−^ mice on day 7 of AIA (Fig. 5E and Fig. S9).

## Discussion

### HS biosynthesis is significantly regulated during macrophage polarization

Both the immune system and HS proteoglycans are complex information networks, in which dynamic interactions between multiple nodes determine biological outcomes. Here, we comprehensively profiled changes in expression of HS biosynthetic machinery during macrophage polarisation, and found that almost two-thirds of the expressed genes were significantly regulated in response to IFNγ + LPS and/or IL4 + IL13, with significant changes in expression of at least one gene from each family of HS modifying enzymes. The most highly regulated gene was the extracellular HS 6-O-sulfatase *Sulf2*, which was downregulated 25-fold by IFNγ + LPS. This extends previous work by Martinez et al. [25], who demonstrated significant changes in expression of HS sulfotransferases during macrophage polarisation, but did not examine HS polymerases (*Ext1*, *Ext2*, *Extl1*, *Extl2*, *Extl3*), epimerase (*Glce*), degrading enzymes (*Hpse*, *Hpse2*) or sulfatases (*Sulf1*, *Sulf2*).

The strong regulation of *Sulf2* expression is striking because SULF1 and SULF2 are the only enzymes that are able to modify HS sulfation in the extracellular environment [13]. N-, 2-O-, 3-O- and 6-O-sulfation are all added to HS during its synthesis in the Golgi apparatus, but once HS is secreted into the extracellular environment, its structure can only be modified by heparanase (encoded by *Hspe*), which cleaves HS into smaller fragments, or by the sulfatases (encoded by *Sulf1* and *Sulf2*), which remove sulfate groups specifically from the 6-carbon position of N-acetylglucosamine residues [40]. SULFs thus carry out the only ‘editing’ of HS that occurs in the extracellular environment, giving these enzymes the unique ability to rapidly fine-tune HS affinity for ligands and so dynamically modulate downstream signaling pathways [43]. It should be noted that no change in overall levels of 6-O-sulfation were observed in some studies of *Sulf2*-null animals [39], suggesting SULF1 and SULF2 may act co-operatively [39], or that SULF2 may may target micro-regions of HS with functional importance.

### *Sulf2*-deficient macrophages have an elevated inflammatory phenotype

The marked reduction in *Sulf2* expression in response to treatment with IFNγ + LPS suggests that SULF2 may have an anti-inflammatory or regulatory role in macrophages. We found that *Sulf2*^+/−^ macrophages exhibited a generally more inflammatory phenotype in vitro, with higher expression of *Tnf*, and lower expression of *Socs3*, *Arg1*, *Cd206* and *Fizz* in response to polarising cytokines. This is in agreement with Zhang et al. [44], who showed that loss of SULF2 expression in bladder cancer cells promoted polarisation of co-cultured THP-1 cells towards an inflammatory phenotype. Further in vitro investigations showed that no evidence that macrophage *Sulf2*-deficiency altered TLR signaling, inflammasome activation, or antigen presentation, so we sought to investigate the role of myeloid *Sulf2* on inflammation in vivo.

*Sulf2* is widely expressed [40], so we adopted a bone marrow transfer approach, to generate chimeric mice with heterozygous deficiency of *Sulf2* in the myeloid lineage. We observed perinatal lethality of *Sulf2*^−/−^ animals, as has been reported for some other *Sulf2*^−/−^ lines [38, 39], but not others [45, 46]. Such strain-dependent effects have also been reported for mice lacking other HS biosynthetic enzymes, such as *Hs3st1* [47] and *Hs6st1* [48], suggesting HS biosynthesis is strongly influenced by modifier genes.

*Sulf2*^+/−^ bone marrow chimeric mice showed significantly increased joint swelling and histological damage in the resolution phase of the macrophage- [49, 50]- and CD4^+^ T cell-dependent [51] antigen-induced arthritis model. This was accompanied by an increased abundance of Th17 cells in joints of *Sulf2*^+/−^ chimeras, consistent with the known ability of this subset to exacerbate inflammation in this [52] and other [53] models of arthritis.

### Increased type I interferon signaling in *Sulf2*-deficient macrophages

To understand how *Sulf2*-deficiency in macrophages could promote Th17 differentiation, we conducted bulk RNA sequencing of cells isolated from AIA joints during the resolution phase of joint inflammation. This showed that differentially expressed genes were related to viral infection responses, suggesting altered type I interferon signaling, with 56% of the differentially expressed genes being known type I interferon-responsive genes. In vitro analyses confirmed elevated responses to IFNβ in *Sulf2*^+/−^ BMDMs, with increased STAT1 phosphorylation and increased induction of *Ccl5*, *Ccl7*, *Tlr3* and *Il6* expression. STAT1 phosphorylation was also increased in vivo. Increased IL6 expression would lead to the observed increase in Th17 abundance and Th7/Threg ratio [54]. This proposed mechanism is consistent with a previous study in lung carcinoma cells, where siRNA or epigenetic silencing of SULF2 was found to activate expression of interferon-inducible genes [55].

Considering the molecular mechanism by which SULF2 could reduce type I interferon signaling, Gordts *et a*l. [28] previously showed that IFNβ signaling in macrophages is modulated by cell surface HS. IFNβ binds strongly to HS (*K*_i_ of 1.4 nM) [28, 56], while IFNα4, interferon-alpha/beta receptor alpha chain (IFNAR1) and interferon-alpha/beta receptor beta chain (IFNAR2) do not [28], and as with all HS ligands, IFNβ/HS affinity is likely to be modulated by the overall level and pattern of HS sulfation. N-sulfation of HS was shown to reduce IFNβ signaling and to be protective in models of atherosclerosis and obesity [28]. Our data here suggest that 6-O-sulfation of HS has an opposite effect, promoting IFNβ signaling and leading to increased *Il6* expression, which promotes the generation of Th17 cells and exacerbates inflammatory arthritis.

Further experiments are required to test the molecular mechanism by which HS alters IFNβ signaling, and we suggest the following model to stimulate such investigations. We propose that N-sulfation of HS could promote IFNβ binding to cell surface HS proteoglycans in preference to its cognate receptors, while 6-O-sulfation generates a different structural motif that may promote formation and/or stabilisation of IFNβ/receptor complexes. This model predicts that homeostatic expression of SULF2 would keep 6-O-sulfation of cell surface HS at low levels, leading to accumulation of IFNβ on N-sulfated HS proteoglycans and keeping IFNβ signaling in check, while in inflammatory environments (or *Sulf2*-deficiency, which mimics and/or exacerbates this), reduced SULF2 would lead to increased HS 6-O-sulfation and elevated IFNβ signaling. This suggests that IFNβ signaling would be further elevated in macrophages deficient for both *Sulf2* and *Ndst1*.

Our findings suggest it is worth investigating whether *Sulf2* could also be protective in other autoimmune conditions where IFNβ signaling promotes Th17-driven inflammation e.g. psoriasis, systemic lupus erythematosus, Sjögren’s syndrome, and neuromyelitis optica [57]. However, IFNβ signaling can also suppress auto-immune responses, such as in relapsing remitting multiple sclerosis, where IFNβ is used therapeutically to slow disease progression [57], and *Sulf2* may have deleterious effects in such contexts. Indeed, Saraswat et al. [58] showed that *Sulf2* expression is elevated in multiple sclerosis lesions, and propose that it contributes to an inhibitory microenvironment that limits remyelination. HS sulfation in numerous tissues and cells types is known to change with ageing [15–17], inflammation [59], infection [60] and metabolic state [18, 61], suggesting a novel mechanism by which co-morbidities could modulate physiological, pathological and therapeutic type I interferon signaling. Establishing what regulates *Sulf2* expression and HS structure more broadly is thus likely to be of importance for multiple auto-immune conditions.

### Further considerations

Looking beyond type I interferon signaling, *Sulf2*-deficiency is also likely to impact the biological activity of multiple other HS-binding proteins, with experimental identification of an affected pathway dependent on the model analysed and its molecular drivers. For example, *Sulf2*-deficient mice have worse outcomes in models of osteoarthritis [62], myocardial infarction [63], and bleomycin-induced lung injury [64], which has been attributed to impaired bone morphogenetic protein [62] and vascular endothelial growth factor [63] signaling and elevated fibroblast growth factor 2 [62] and TGFb [64] signaling.

Both cell-associated [65–67] and secreted [40, 63, 66] forms of SULF2 have been described, suggesting the enzyme can act in both an autocrine and paracrine manner. Recently, El Masri et al. [68] found that SULF2 localisation and activity can be modulated by post-translational modification, with attachment of a chondroitin or dermatan sulfate glycosaminoglycan chain to the enzyme’s HD domain increasing cell surface association and reducing 6-O-sulfatase activity. Degradation of the glycosaminoglycan chain by hyaluronidase reduced cell surface binding and increased SULF2 activity [68]. Conditions that increase hyaluronidase expression could thus switch SULF2 from autocrine to enhanced paracrine activity. Our in vivo data are likely to reflect autocrine activity of SULF2, because *Sulf2*-deficiency in myeloid cells was not compensated for by expression in other cells of the joint, such as synovial fibroblasts, which express abundant *Sulf2* in RA [69]. Intriguingly, Siegel et al. [69] found that SULF2 expressed by rheumatoid synovial fibroblasts promotes inflammation, by increasing transcriptomic and phenotypic responses of these cells to TNF. This suggests that synovial fibroblast and myeloid SULF2 have opposing effects on joint inflammation, with synovial SULF2 potentially contributing to initiation of inflammation, and myeloid SULF2 promoting resolution. Further investigation is required to define the kinetics of SULF2 expression and activity in these cell types.

### Electronic supplementary material

Below is the link to the electronic supplementary material.

Supplementary Material 1

Supplementary Material 2

## Data Availability

The datasets used and analysed during the current study are available from the corresponding author on reasonable request.

## Abbreviations

AIA: Antigen-induced arthritis
APRIL: A Proliferation-Inducing Ligand
BMDMs: Bone marrow-derived macrophages
BMDCs: Bone marrow-derived dendritic cells
DCs: Dendritic cells
FBS: Fetal bovine serum
FSL-1: Fibroblast-stimulating lipopeptide-1
FMO: Fluorescence minus one
GM-CSF: Granulocyte-macrophage colony-stimulating factor
HS: Heparan sulfate
IFN: Interferon
IFNAR1: Interferon-alpha/beta receptor alpha chain
IFNAR2: Interferon-alpha/beta receptor beta chain
KEGG: Kyoto Encyclopedia of Genes and Genomes
LPS: Lipopolysaccharide
mBSA: Methylated bovine serum albumin
M-CSF: Macrophage colony-stimulating factor
MFI: Mean fluorescence intensity
MOI: Multiplicity of infection
OVA: Ovalbumin
Pam3CSK4: N-palmitoyl-S-[2,3-bis(palmitoyloxy)-(2RS)-propyl]-[R]-cysteinyl-[S]-seryl-[S]-lysyl-[S]-lysyl-[S]-lysyl-[S]-lysine
PCA: Principal component analysis
PMA: Phorbol 12-myristate 13-acetate
Poly (I:C): Polyinosinic Polycytidylic acid
RPMI: Roswell Park Memorial Institute 1640 medium
SULF2: Sulfatase 2
TLDA: TaqMan Low-Density Array
TLR: Toll-like receptor
TGFβ: Transforming growth factor beta
WT: Wild-type
