# Supplementary material for: The extracellular heparan sulfatase SULF2 limits myeloid IFNβ signaling and Th17 responses in inflammatory arthritis
Source: Cell Mol Life Sci. 2024 Aug 14;81(1):350. doi: 10.1007/s00018-024-05333-w (PMC11335274; doi:10.1007/s00018-024-05333-w)
Supplement: Supplementary file 1 — Supplementary Material 1 [file 18_2024_5333_MOESM1_ESM.pdf]

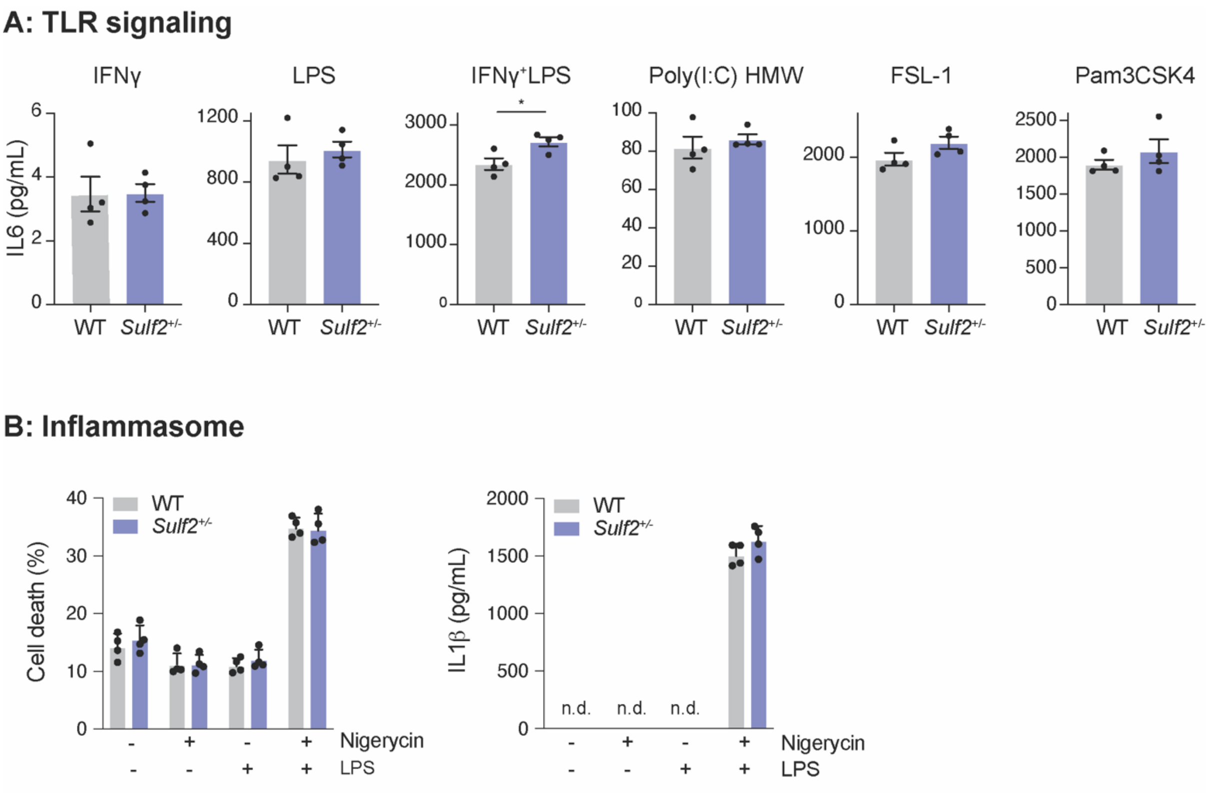

Supplementary Fig. S1. TLR signaling and inflammasome activation were unchanged in *Sulf2*$^{+/-}$ BMDMs. (**A**). BMDMs from WT and *Sulf2*$^{+/-}$ mice were stimulated for 6 h with IFNγ, LPS, IFNγ+LPS (all at 100 ng/ml), FSL-1 (100 ng/ml), poly(I:C) (10 ng/ml) or Pam3CSK4 (100 ng/ml) and IL6 secretion was measured by ELISA (n=4, mean ± SEM, analysed by a two-tailed Student's T-test. *, P<0.05). (**B**). BMDMs from WT and *Sulf2*$^{+/-}$ mice were stimulated with LPS (100 ng/ml, 6 h) with nigericin (5 μM) added for the last 2 h of culture. Cell death and IL1β secretion were quantified (n=4, mean ± SEM, analysed by two-way ANOVA with Bonferroni's correction for multiple comparisons).

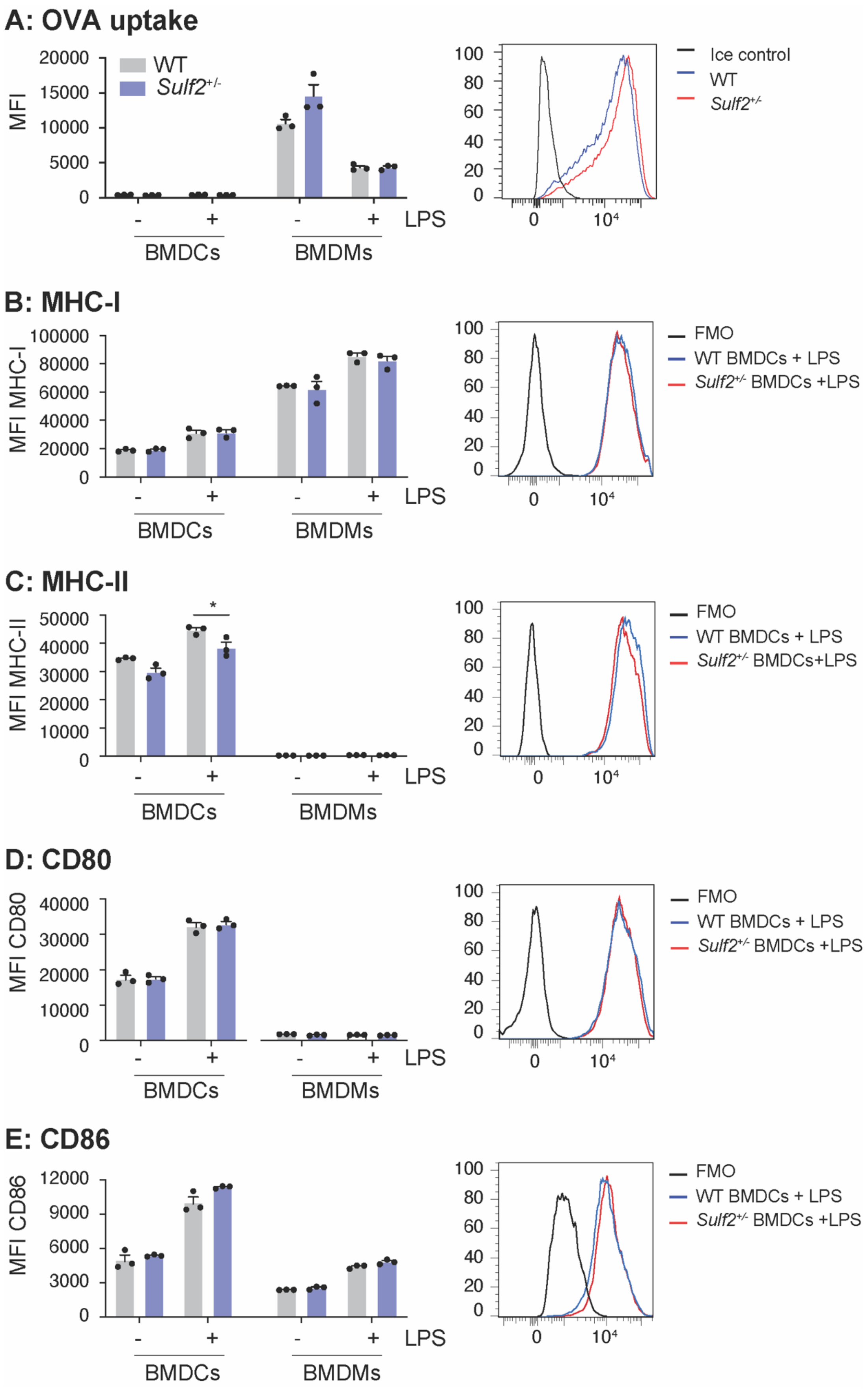

Supplementary Fig. S2. OVA uptake and expression of co-stimulatory molecules was unchanged in *Sulf2*$^{+/-}$ macrophages. Bone marrow cells from WT and *Sulf2*$^{+/-}$ mice (n=3) were cultured with M-CSF (100 ng/ml, 7 d)

to generate BMDMs, or with GM-CSF (20 ng/ml, 7 d). (**A**). Cells were stimulated with LPS (100 ng/ml, 6 h) and incubated with Alexa647-conjugated OVA (0.1 mg/ml, 1 h, 37 °C) before analysis by flow cytometry. BMDCs in the GM-CSF-generated cell population were analysed by gating for CD11c^{+}MHC-II$^{int\text{-}high}$, followed by gating for CD11b^{int}MHC-II^{high} (n=3, mean ± SEM, analysed by a two-way ANOVA with Bonferroni's correction for multiple comparisons; *, P<0.05). (**B-E**). Cells were stimulated with LPS (100 ng/ml, 6 h) and cell surface expression of MHC-I (**B**), MHC-II (**C**), CD80 (**D**) and CD86 (**E**) measured by flow cytometry using the gating strategy described in (**A**) (n=3, mean ± SEM, analysed by a two-way ANOVA with Bonferroni's correction for multiple comparisons; *, P<0.05).

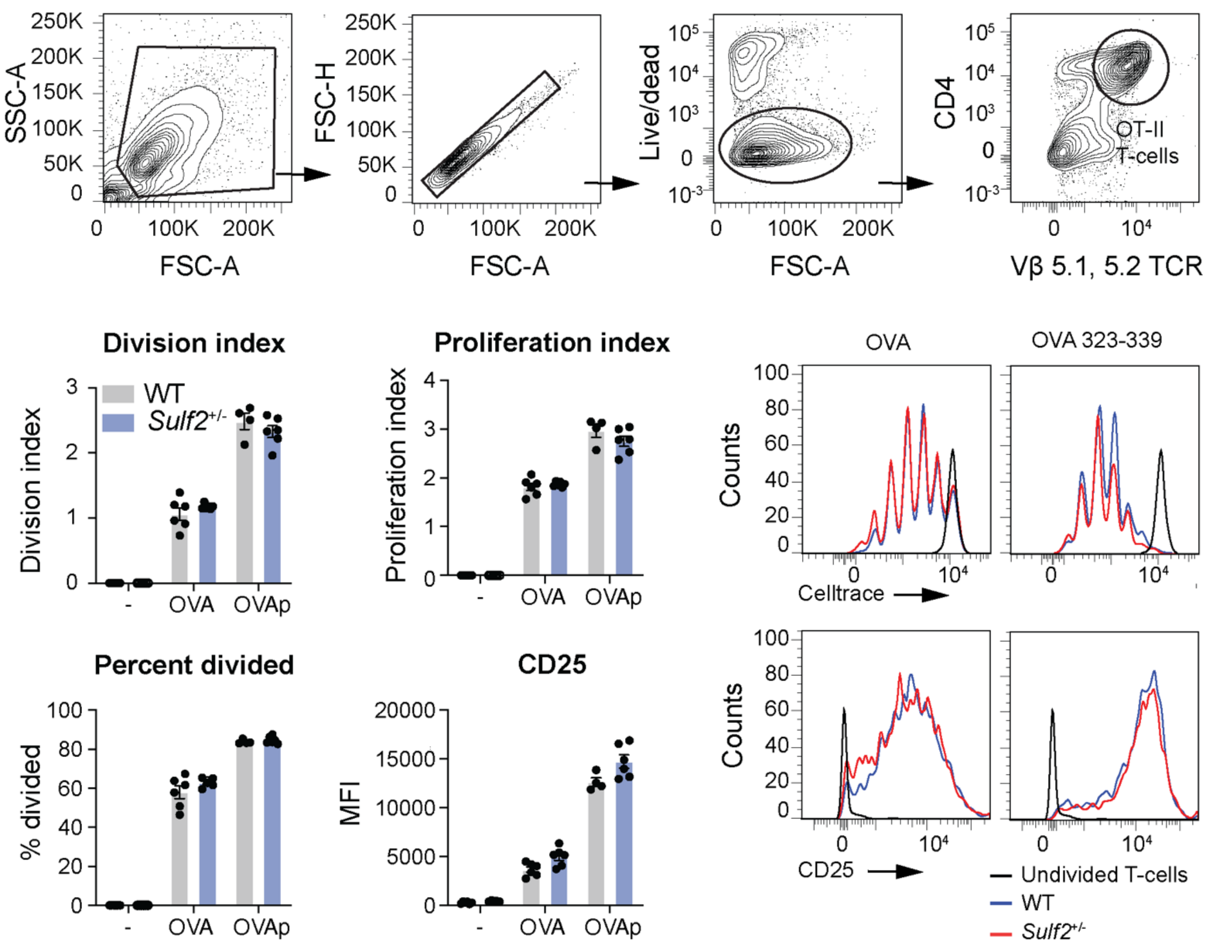

Supplementary Fig. S3. *Sulf2*-deficiency did not affect dendritic cell presentation of OVA antigen *in vitro*. CD4^{+} OT-II T-cells were labelled with CellTrace Violet and co-cultured (4 d) with LPS-activated WT or *Sulf2*$^{+/-}$ BMDCs. OVA was added to antigen-presenting cells before LPS-activation, or OVA peptide 323-339 was added during co-culture with CD4^{+} T-cells. CD4^{+} T-cell proliferation and expression of CD25 were analysed by flow cytometry, using the gating strategy shown in top panel (n=3, analysed in duplicate, mean ± SEM, analysed by a two-way ANOVA with Bonferroni's correction for multiple comparisons).

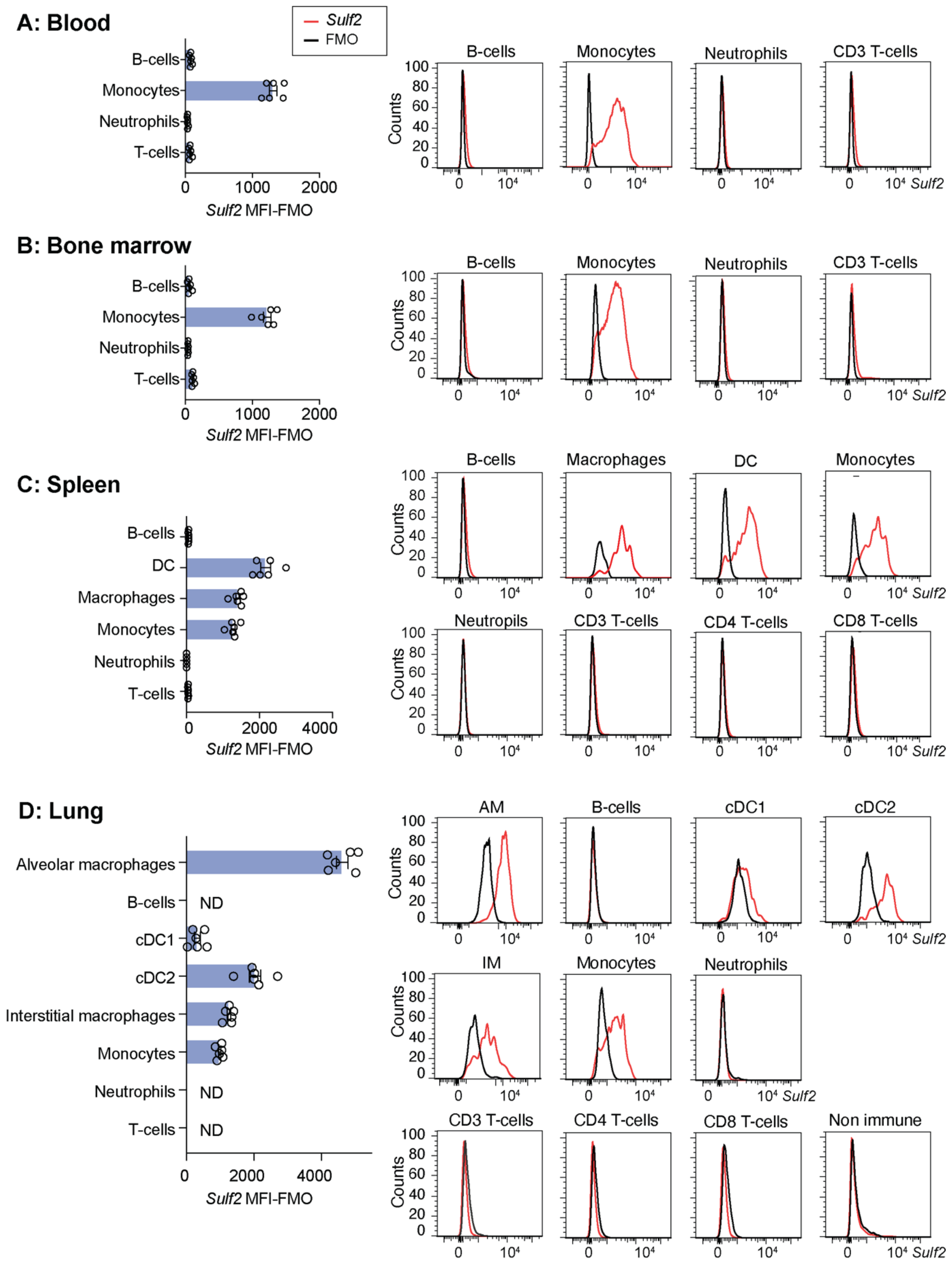

Supplementary Fig. S4. *Sulf2* was highly expressed by monocytes in murine blood and bone marrow, and by macrophages, monocytes and dendritic cells in the spleen and lung. *Sulf2* expression in immune populations was quantified by analysing single cell suspensions from blood, bone marrow, spleen and lung of C57BL/6 mice using the PrimeFlow RNA assay. Fluorescence was analysed by flow cytometry and expressed as the mean fluorescence

intensity (MFI) minus the *Sulf2* fluorescence minus one (FMO). Representative histograms MFI-MFO (± SEM, n=6) are shown.

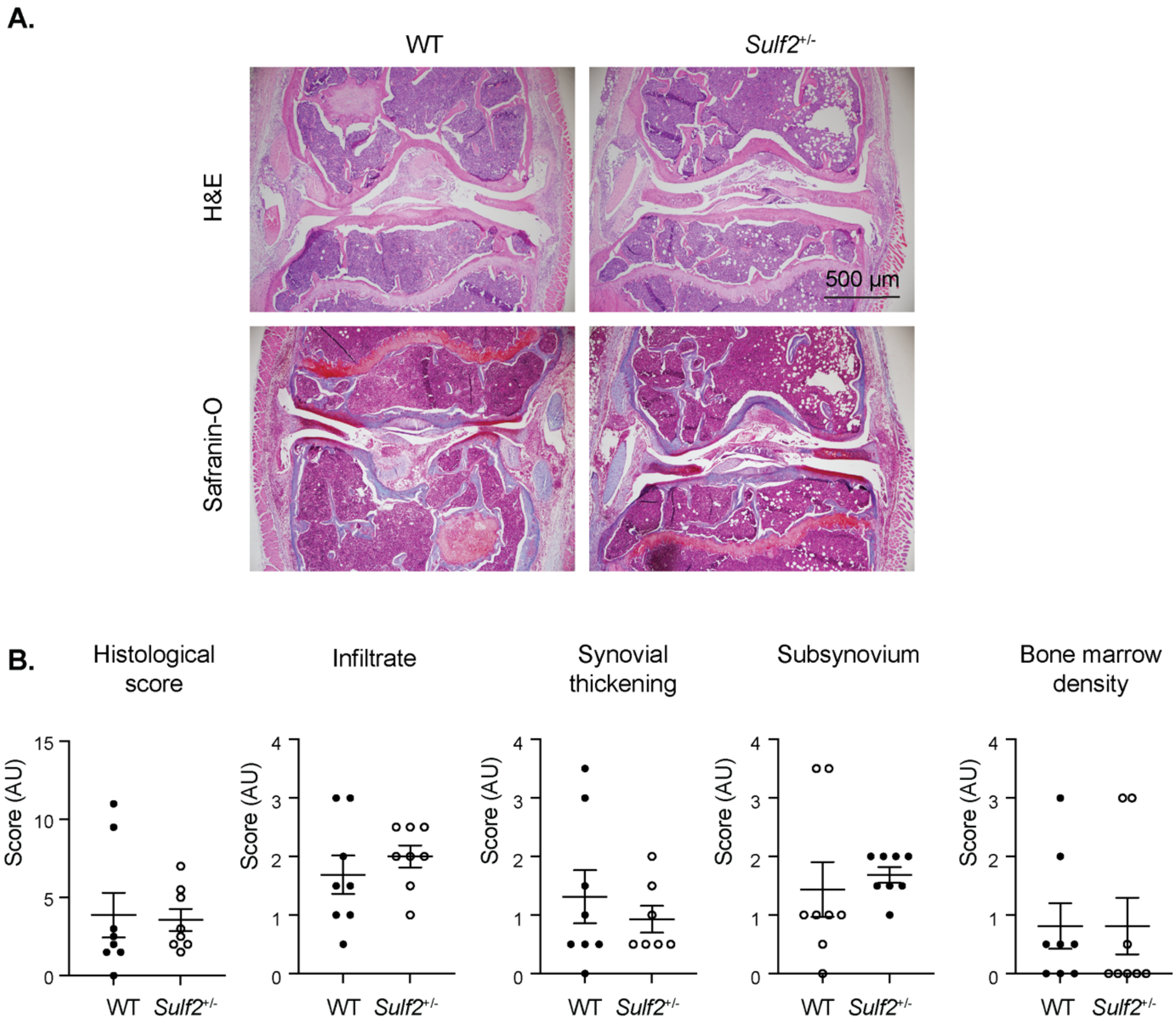

Supplementary Fig. S5. AIA histology scores were not affected at day 2 in *Sulf2*-deficient bone marrow-chimera mice. (**A**). WT and *Sulf2*$^{+/-}$ bone marrow chimeric mice (n=8) were immunised with mBSA (100 µg) in CFA, and arthritis induced 3 weeks later by intra-articular tibiofemoral injection of mBSA (100 µg, right knee). 2 days later, the mice were sacrificed and sections of the knees stained with safranin-O and H&E. (**B**). Histology of the joint (4x magnification) was examined to calculate the total histological score, synovium and subsynovium thickness, and bone marrow density (mean ± SEM, analysed by a two-tailed Mann-Whitney u test).

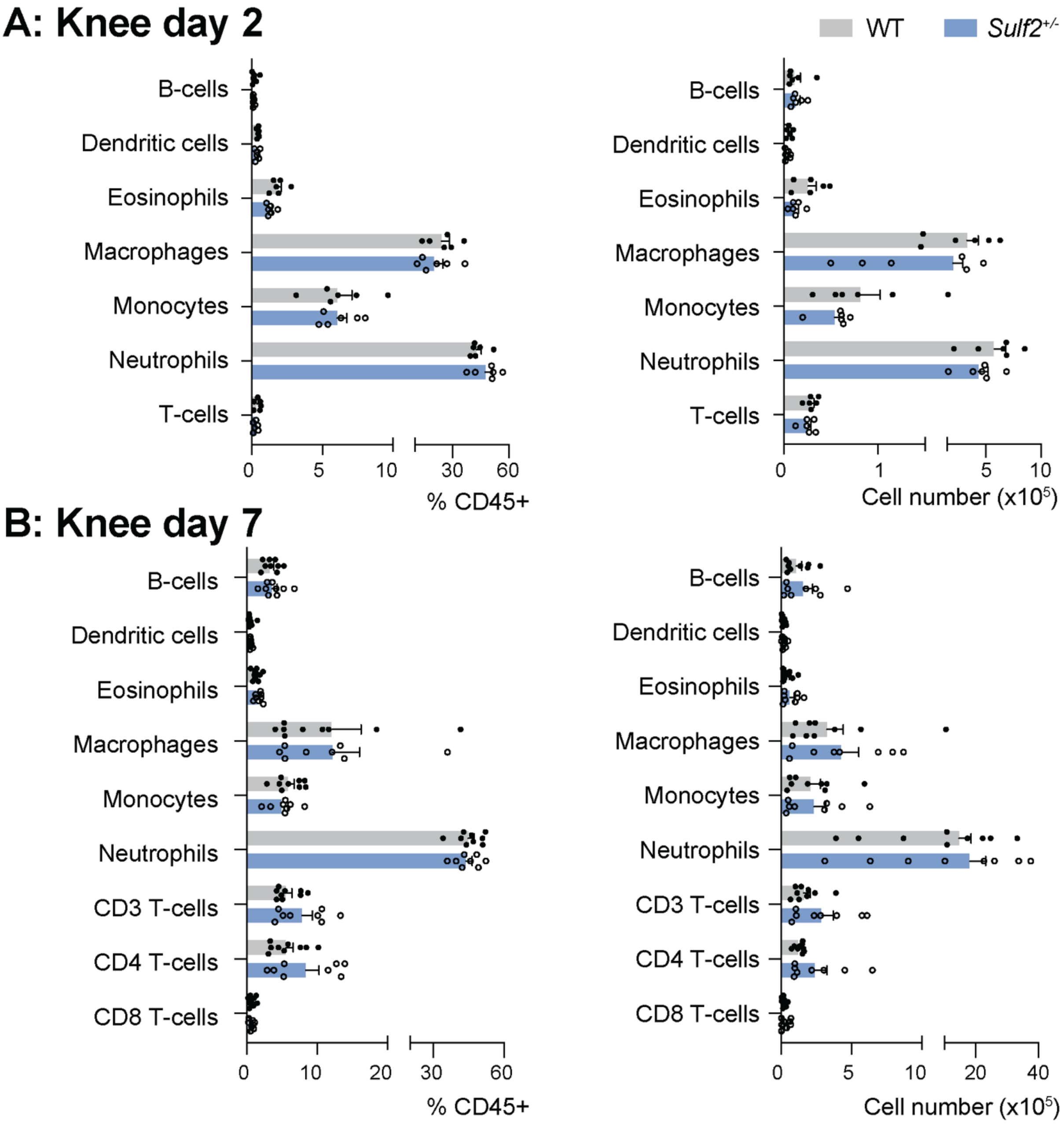

Supplementary Fig. S6. *Sulf2*-deficiency did not alter the abundance or frequency of immune cell types in the knee of AIA mice. WT and *Sulf2*$^{+/-}$ bone marrow chimeric mice were immunised with mBSA (100 µg) and arthritis was induced 3 weeks later by intra-articular tibiofemoral injection of 100 µg mBSA (right knee). 2 days (**A**, n=6) or 7 days (**B**, n=9) later, affected knees were digested and the prevalence of myeloid and adaptive immune cell populations was determined by flow cytometry (mean ± SEM, analysed by multiple unpaired *t*-tests, followed by multiplicity correction using the two-stage step-up method of Benjamini, Krieger and Yekutieli).

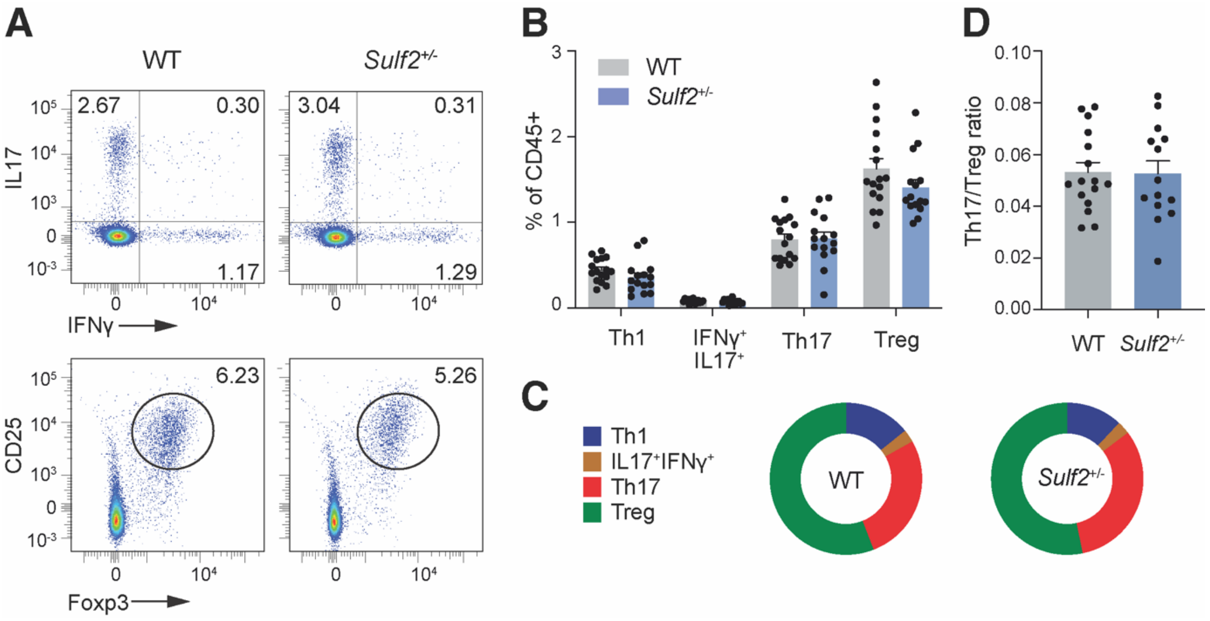

Supplementary Fig. S7. The Th17/Treg ratio in the inguinal lymph nodes of *Sulf2*-deficient mice was unchanged at day 7 of AIA. AIA was induced in WT and *Sulf2*$^{+/-}$ bone marrow chimeric mice (n=16). 7 days later, cells were isolated from the inguinal lymph nodes and stimulated for 4 h with PMA (20 ng/ml) and ionomycin (1 µg/ml) in the presence of protein transport inhibitors. CD3^{+}, CD4^{+} and CD8^{+} T-cell subsets were analysed by flow cytometry. (**A**). Representative dot plots for Th17 (IFNγ^{+}IL17^{+}) and Treg (CD25^{+}Foxp3^{+}) subsets in inguinal lymph nodes. (**B**). The abundance of T cell subsets (as a percentage of CD45^{+}) was calculated (mean ± SEM, analysed by a two-tailed Student's *t*-test). (**C**). Graphical representation of data in (**B**). (**D**). The Th17/Treg ratio was calculated from data in (**B**) (mean ± SEM, analysed by two-tailed Student's *t*-test).

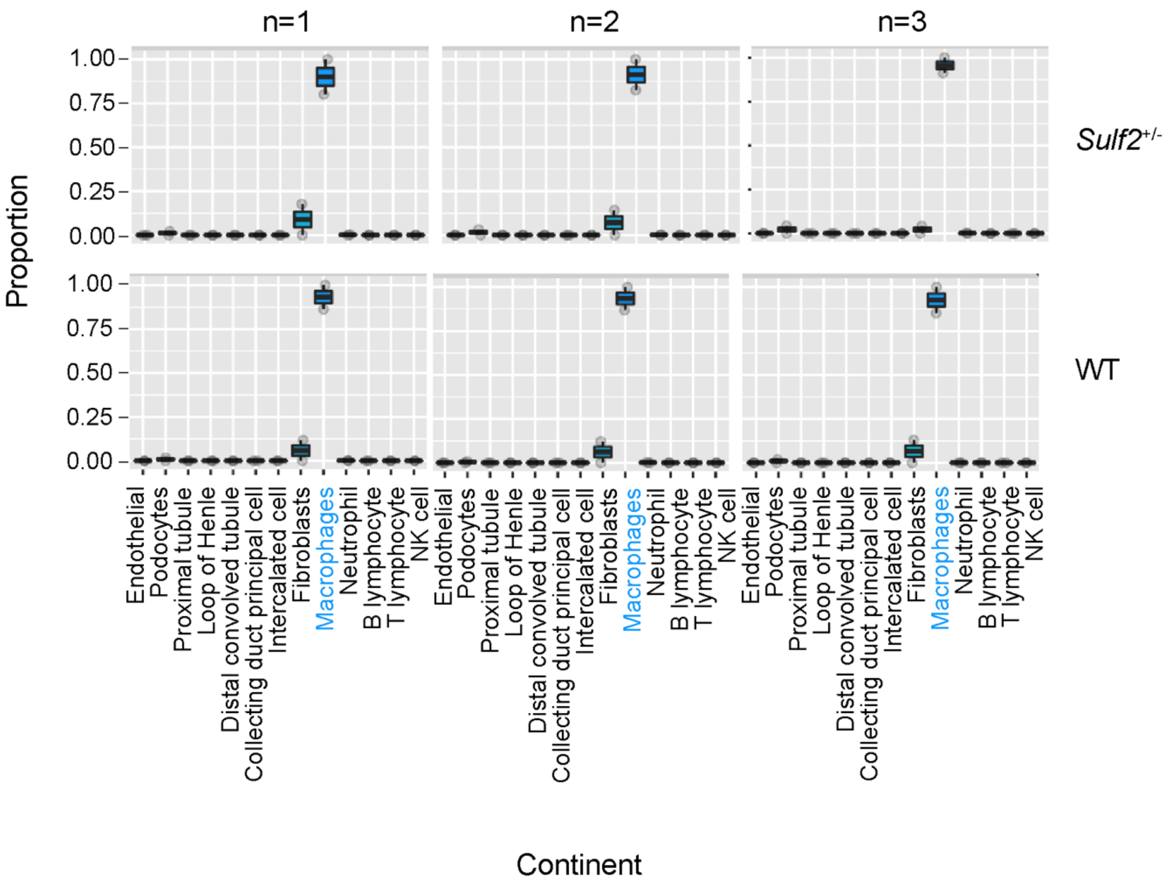

Supplementary Fig. S8. Devolution of bulk RNA sequencing data with MuSIC indicated the majority of transcripts originated from macrophages. The algorithm MuSIC was used to estimate cell type proportions for the bulk RNA sequencing data from *Sulf2*$^{+/-}$ and WT mice (n=3 each) using single cell RNASeq of known cell types [33]. This predicted that the majority (>85%) of cells sequenced were macrophages, with a small proportion of fibroblasts.

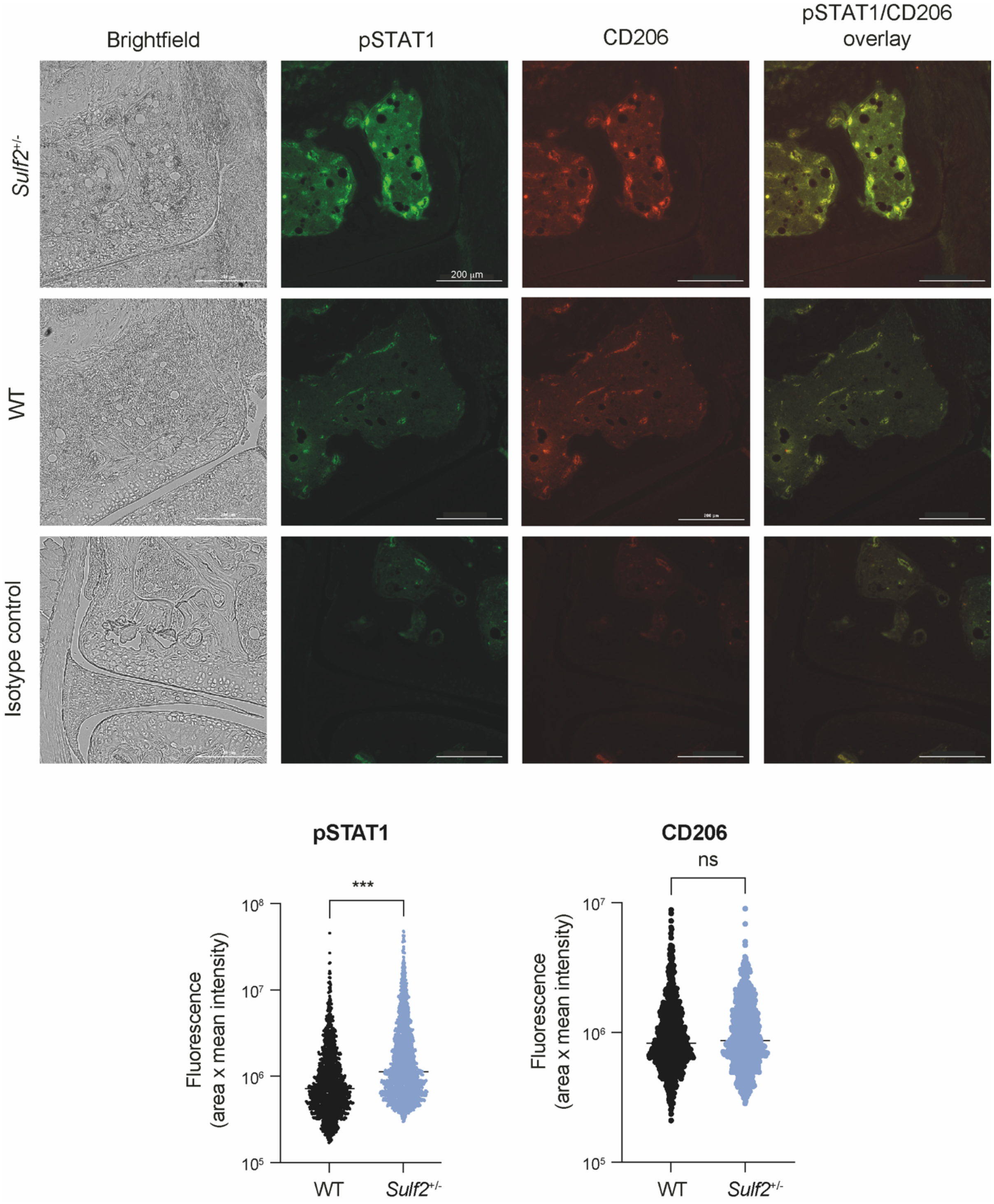

Supplementary Fig. S9. Immunohistochemistry analysis showed increased pSTAT1 staining in knee joints of *Sulf2*-deficient mice at day 7 of AIA. Longitudinal sections from WT and *Sulf2*$^{+/-}$ (n=3 per genotype) bone marrow chimera mice on day 7 of AIA were stained for pSTAT1 and CD206. Representative images are shown, along with quantification of pSTAT1 and CD206 fluorescent signal from at least 700 cell clusters per genotype (analysed by two-tailed Mann-Whitney u test. ***, P<0.001; ns, not significant).

Supplementary Table S1: TaqMan primers used to measure gene expression by RT-qPCR and microfluidic cards.

Target	Assay ID
18s	Mm04205640_g1 (RT-qPCR) and Hs99999901_s1 (TLDA)
Arg1	Mm00475988_m1
Agrn	Mm01545823_g1
Atg7	Mm00512209_m1
Axl	Mm00437221_m1
Cdkn1a	Mm04205640_g1
Cdkn2a	Mm00494449_m1
Elane	Mm00469310_m1
Ext1	Mm01194490_m1
Ext2	Mm00468775_m1
Extl1	Mm00517161_m1
Extl2	Mm00469621_m1
Extl3	Mm01317041_m1
Gapdh	Mm03302249_g1 (RT-qPCR) and Mm99999915_g1 (TLDA)
Gas6	Mm00490378_m1
Glce	Mm004736607_m1
Gpc1	Mm01290371_m1
Gpc2	Mm00549653_g1
Gpc3	Mm00516722_m1
Gpc4	Mm00515035_m1
Gpc5	Mm01235111_m1
Gpc6	Mm00516232_m1
Hpse	Mm004617608_m1
Hpse2	Mm011985307_m1
Hs2st1	Mm00478684_m1
Hs3st1	Mm019640308_s1
Hs3st2	Mm00616933_m1
Hs3st3a1	Mm007809007_s1
Hs3st3b1	Mm030529707_s1
Hs3st4	Mm03649146_m1
Hs3st5	Mm01192940_m1
Hs3st6	Mm01299930_m1
Hs6st1	Mm0122968_s1 (RT-qPCR) and Mm012296908_s1 (TLDA)
Hs6st2	Mm00479296_m1
Hs6st3	Mm01225470_s1
Hspg2	Mm01181173_g1

Ifnγ	Mm01168134_m1
Il1b	Mm00434228_m1
Il6	Mn00446190_m1
Il10	Mn01288386_m1
Il12b	Mm00434174_m1
Il17a	Mm00439619_m1
Il23a	Mn01160011_g1
Itgb3	Mm004439880_m1
Mertk	Mm00434920_m1
Mfge8	Mm00500549_m1
Mrc1	Mm01329362_m1
Mpo	Mm01298424_m1
Ndst1	Mm00447005_m1
Ndst2	Mm004478108_m1
Ndst3	Mm00453184_m1
Ndst4	Mm004807607_m1
Nos2	Mm00440489_m1
Pparg	Mm00440940_m1
Pros1	Mm01343426_m1
Retnla	Mm00445109_m1
Rplp0	Mm00725448_s1
Sdc1	Mm00448918_m1 (RT-qPCR) and Mm012758609_m1 (TLDA)
Sdc2	Mm00484718_m1 (RT-qPCR) and Mm04207492_m1 (RT-qPCR, TLDA)
Sdc3	Mm01179833_m1
Sdc4	Mm00488527_m1
Socs3	Mm00545913_s1
Stat1	Mm01257286_m1
Stat6	Mm01160477_m1
Sulf1	Mm00552283_m1
Sulf2	Mm01248029_m1
Tgfb1	Mn01178820_m1
Tnf	Mn00443258_m1
Tgfbr3	Mm008035308_m1
Tyro3	Mm00444547_m1

Supplementary Table S2: Sybr primers used to measure gene expression by RT-qPCR.

Gene	Forward primer	Reverse primer
Ccl5	AGGAGTATTTCTACACCAGC	CAGGGTCAGAATCAAGAAAC
Ccl7	CTCTCTCACTCTCTTTCTCC	TCTGTAGCTCTTGAGATTCC
Gapdh	TGACCACAGTCCATGCCATC	GACGGACACATTGGGGGTAG
Il6	AAGAAATGATGGATGCTACC	GAGTTTCTGTATCTCTCTGAAG
Tlr3	AATAGCATCAAAAGAAGCCG	GATGTACCTTGAATCTTCTGC

Supplementary Table S3: Commercial antibodies used in flow cytometry
.

Target	Host species	Isotype	Clone	Company
CCR7	Rat	IgG2a, κ	4B12	BioLegend
CD3	Hamster	IgG1, κ	145-2C11	BD Biosciences
CD4	Rat	IgG2a, κ	RM4-5	eBioscience
CD8	Rat	IgG2a, κ	53.6-7	BioLegend
CD11b	Rat	IgG2b, κ	M1/70	BioLegend
CD11c	Hamster	IgG1, λ2	HL3	BD Biosciences
CD19	Rat	IgG2a, κ	ID3/CD19	BioLegend
CD25	Rat	IgG1, λ	PC61	BioLegend
CD36	Armenian hamster	IgG	MH36	BioLegend
CD40	Rat	IgG2a, κ	3/23	BioLegend
CD45	Rat	IgG2b, κ	30-F11	BioLegend
CD80	Hamster	IgG	16-10A1	BioLegend
CD86	Rat	IgG2a, κ	GL1	BD Pharmingen
CD169	Rat	IgG2a, κ	3D6.112	BioLegend
CD204	Human	IgG1	REA148	Miltenyi Biotech
CD206	Rat	IgG2a, κ	C068C2	BioLegend
F4/80	Rat	IgG2a, κ	BM8	BioLegend
Foxp3	Rat	IgG2a, κ	FJK-16s	eBioscience
IFNγ	Rat	IgG1, κ	XMG1.2	BioLegend
IL17	Rat	IgG1, κ	TC11- 18H10.1	BD Pharmingen
Ki-67	Rat	IgG2a, κ	16A8	BioLegend
Ly6C	Rat	IgG2c, κ	HK1.4	BioLegend
Ly6G	Rat	IgG2a, κ	1A8	BD Biosciences
Marco	Mouse	IgG2a, κ	F-3	Santa Cruz
Mertk	Rat	IgG2a	FAB5912P	R&D Systems
MHC-I	Hamster	IgG	Sf1-1.1	BD Biosciences
MHC-II	Rat	IgG2b, κ	M5/114.15.2	BD Biosciences
Rorγt	Rat	IgG2a, κ	AFKJS-9	eBioscience

Supplementary Table S4 – see separate Excel file: Significantly differentially expressed genes. Bulk RNA sequencing was conducted on cells isolated from knees of WT and *Sulf2*$^{+/-}$ chimeric mice 7 days after initiation of AIA, and differential gene expression analysis performed using DeSeq2.
